# Supplementary material for: Do monetary incentives encourage local communities to collect and upload mosquito sound data using smartphones? A case study in the Democratic Republic of the Congo
Source: PLoS One. 2025 Aug 7;20(8):e0314122. doi: 10.1371/journal.pone.0314122 (PMC12331038; doi:10.1371/journal.pone.0314122)
Supplement: S2 File — (PDF) [file pone.0314122.s002.pdf]

SUPPORTING INFORMATION: S2 Appendix

Do monetary incentives encourage local communities to collect and upload mosquito sound data using smartphones? A case study in the Democratic Republic of the Congo

Storer *et al.*

This is the S2 Appendix of supporting information. The overall supplementary information contains the DHS Demographic Survey (*Supplement 1 Appendix*) and the English version of the focus group discussion questions (***Supplement 2 Appendix***). *Supplement 3 Table* shows results from comparisons of the demographic survey between study provinces, trial groups overall, and trial groups within each province using Wilcoxon rank sum tests or Fisher's Exact Test depending on sample group size and data type.

S2 Appendix: Focus Group Discussions: Pre and Post Trial

HumBug: Development of a mosquito surveillance tool for least developed countries Focus group/interview guide to engage community members in rural DRC

Fully Group Discussion/Interview Session Duration 80 mins

Including, completing 10 mins consent forms (completed individually)

Ensure all consents are correctly completed and begin registration

Including, completing the 15 mins demographic questionnaire (completed individually)

[Begin the group by introducing yourself, indicating the purpose of the group and the history of the search.]
10 minutes

For example: Hello, my name is ___ and I am a researcher at the University of Oxford. There is also xxx in the room who will help me in this discussion. We've brought you here today because we want to hear your thoughts on a new mosquito survey tool (HumBug) we've developed. We are developing an acoustic sensor that identifies different species of mosquitoes by the sound they emit while flying. It is available as an app used on smartphones. With your permission, we plan to test our sensors in your village, and invite you to help us in our work. We need to place our acoustic sensors on your mosquito nets to listen for mosquitoes that approach you during the night. We will provide the volunteers with a new mosquito net with a pocket where we will place the mosquito sensor (one smartphone - one per house). As part of the study, you will need to turn on the sensor before going to bed or under your mosquito net and check and charge it if necessary. Otherwise, its presence will have no impact on your daytime or nighttime activity. The sensor will record all sounds (not just mosquitoes) from 6 p.m. to 6 a.m. In the morning, we will ask you to upload the captured sound data to a web platform that we will provide to you. Automated algorithms will then detect any voice activity captured in the recordings and delete it at that time without any human intervention. We will also need to collect the geolocation of your sensors in order to obtain maps of mosquitoes in particular locations (called "heat maps"). We will collect your approximate locations, such as street or general area within a radius of 50 to 100 meters. We will not locate your homes. We'd like to ask for the location of the sensors through a Google app, in case the sensors get lost during field testing. This will be done to preserve your privacy by remotely wiping user data from the sensors.

The sampling will be conducted over a four-month period that includes the peak of the mosquito season, from 6 p.m. to 6 a.m.

Our long-term goal for the project is for communities to maintain and support the use of the app themselves through a network of community volunteers. So we're working to understand the kind of incentives that would make becoming a community volunteer attractive and the ethical considerations that might come with it.

Therefore, we plan to conduct a randomized controlled trial (RCT) in which a household member who agrees to participate in the study will be randomized to one of the different study arms in each of the three trial groups and the control group. The four groups are as follows: (a) a monetary incentive, in the form of an airtime credit, will be transferred to participants' personal cell phones each time they upload mosquito noise data on the days they are scheduled to do so; (b) SMS reminders will be sent to participants' personal mobile phones on days they are scheduled to collect mosquito noise data. The text message will ask participants to activate and place their sensors in their net pockets before going to bed on those nights; (c) monetary incentive and text message reminders will be sent to entrants' personal phones; (d) no intervention. The RCT will take place over four months.

[Establish ground rules]

There are a couple of things I'd like to touch on before we begin. First of all, there are no right or wrong answers, but rather different points of view. We'd like you to feel free to share your point of view, even if it differs from that of others. Sometimes someone in the group has a strong opinion about something and you have the exact opposite opinion. We'd love to hear what you have to say.

Remember, we care about negative feedback just as much as we care about positive feedback, and sometimes negative feedback is the most helpful.

As we record this discussion, please speak louder and try not to speak over others.

We'll only use first names here, and in our future reports, there won't be any names attached to comments.

My role here is to ask questions, listen, and help facilitate discussion. Feel free to talk to each other. In these discussions, some people tend to talk a lot and others don't say much. But it's important for us to hear from each of you today because you have different experiences. So I might ask for someone's opinion if they don't talk a lot and move the discussion to someone else if someone talks a lot, to make sure we're hearing everybody.

We have limited time today and we have a lot of things to look at, so if anyone shares something that is interesting, but not directly related to the issues that we are discussing, I might suggest that we leave the idea aside, I will take note, and if there is time left at the end, we can come back to it.

We'll finish around xxx.

[Icebreaker, we usually ask people to walk around the room introducing themselves by their first name and saying what they like to do on the weekends, starting with yourself and staff members and then going around the group members.]

Start.

Prior to the start of the RCT:

FOCUS GROUP SCHEDULE/INTERVIEW GUIDE (40 minutes)

1. **Do you understand the purpose of this study?**
2. **Do you understand how we are going to install the smartphone in your mosquito net?**
3. **Do you understand how you might be assigned to a trial group, if you were to participate in the study?**
4. **What would motivate you to participate in the study and why?**
5. **What help/support do you think you will need to use the smartphone at home, as part of the trial?**
6. **Can you identify any potential challenges in conducting the trial at your home? What do you think are the solutions?**
7. **What impact would the trial have on your daily life?**
8. **Would you like us to update you on the progress of the RCT and, at the end of the trial, give you an overview of the results?**

Following the completion of the RCT, the research team will provide community members with an overview of the results:

9. **Are you surprised by the results?**
10. **What did you think of the smartphone left at home?**
    Proposal : Strengths or weaknesses. Was the new mosquito net helpful?
11. **Did the experience have an impact on your daily life?**
    Proposal : Were there any problems turning on the smartphone before going to bed/under the mosquito net, checking it, charging it and downloading the data from the smartphone?
12. **(For those who received financial incentives): Was the amount enough?**
13. **Is there anything you wish we had done differently during this trial?**
14. **Would you participate in a similar study again in the future? If so, why? If not, why not?**

Closing (5 minutes)
Ask them if they have any final questions (Answer the questions). Thank the participants
