# Supplementary material for: Do monetary incentives encourage local communities to collect and upload mosquito sound data using smartphones? A case study in the Democratic Republic of the Congo
Source: PLoS One. 2025 Aug 7;20(8):e0314122. doi: 10.1371/journal.pone.0314122 (PMC12331038; doi:10.1371/journal.pone.0314122)
Supplement: S3 File — (PDF) [file pone.0314122.s003.pdf]

SUPPORTING INFORMATION: S3 Table

Do monetary incentives encourage local communities to collect and upload mosquito sound data using smartphones? A case study in the Democratic Republic of the Congo

Storer *et al.*

This is the S3 Table of supporting information. The overall supplementary information contains the DHS Demographic Survey (*Supplement 1 Appendix*) and the English version of the focus group discussion questions (*Supplement 2 Appendix)*. ***Supplement 3 Table*** shows results from comparisons of the demographic survey between study provinces, trial groups overall, and trial groups within each province using Wilcoxon rank sum tests or Fisher's Exact Test depending on sample group size and data type.

Supplement 3: Table of Demographic Comparisons

Compared Groups					
Test Type	Compared Trait	Bandundu (n=74) and Kinshasa (n=68)	Control (n=74) and Incentive (n=68)	Kinshasa	Bandundu
				Control (n=37) and Incentive (n=31)	Control (n=37) and Incentive (n=37)
Wilcoxon rank sum test with continuity correction	Age	W = 2383, p-value = 0.35	W = 1843, p-value = 0.27	W = 381, p-value = 0.16	W = 428, p-value = 0.48
		95% CI [-2, 7]	95% CI [-7, 2]	95% CI [-11, 2]	95% CI [-9, 4]
	Income	W = 2186.5, p-value = 0.32	W = 1722.5, p-value = 0.41	W = 299.5, p-value = 0.061	W = 446, p-value = 0.98
		95% CI [-20000, 80000]	95% CI [-90000, 20000]	95% CI [-1.70e+05, 5.15e-11]	95% CI [-100000, 70000]
	Years in Home	W = 2213.5, p-value = 0.84	W = 2230.5, p-value = 0.34	W = 480.5, p-value = 1	W = 538, p-value = 0.31
		95% CI [-1.88e-11, 1.04e-11]	95% CI [-2.26e-12, 3.80e-11]	95% CI [-2.38e-12, 2.38e-12]	95% CI [-7.94e-11, 8.65e-12]
	Number of Adults in Home	W = 1680, p-value = 0.017	W = 2110, p-value = 0.87	W = 510, p-value = 0.67	W = 517.5, p-value = 0.55
		95% CI [-1.00e+00,-3.50e-11]	95% CI [-6.75e-11, 1.81e-11]	95% CI [-1.35e-11, 1.00]	95% CI [-7.05e-11, 4.06e-11]
	Number of Children in Home	W = 2232.5, p-value = 0.80	W = 2576, p-value = 0.017	W = 553.5, p-value = 0.30	W = 599.5, p-value = 0.084
		95% CI [-1, 1]	95% CI [4.77e-11, 2.00]	95% CI [-1, 2]	95% CI [-2.53e-11, 2.00]
	Use a Bank	p-value = 0.017	p-value = 1	p-value = 1	p-value = 0.14
		95% CI [0.072, 0.82]	95% CI [0.29, 2.79]	95% CI [0.12, 4.93]	95% CI [0.042, 1.63]
**Fisher's Exact Test for Count Data**	Sex	p-value = 1	p-value = 0.35	p-value = 1	p-value = 1
		95% CI [0.25, 3.48]	95% CI [0.50, 7.42]	95% CI [0.17, 6.39]	95% CI [0.081, 9.10]
	Marital Status	p-value = 0.014	p-value = 0.64	p-value = 0.23	p-value = 0.52
	Education Level	p-value = 0.11	p-value = 0.24	p-value = 0.97	p-value = 0.25
	Profession	p-value = 0.26	p-value = 0.044	p-value = 0.43	p-value = 0.26

Fishers Exact Test for Count Data	Use a Smartphone to Bank	p-value = 0.23	p-value = 0.20	p-value = 0.16	p-value = 1
		95% CI [0.63, 5.96]	95% CI [0.71,7.16]	95% CI [0.58 ,18.31]	95% CI [0.13, 4.57]
	Subsistence Farmers	p-value = 0.80	p-value = 0.80	p-value = 1	p-value = 0.45
		95% CI [0.28, 2.56]	95% CI [0.26, 2.54]	95% CI [0.16, 4.46]	95% CI [0.072, 2.68]
	Own Land	p-value = 0.89	p-value = 0.89	p-value = 1	p-value = 1
	Livestock	p-value = 0.18	p-value = 0.19	p-value = 1	p-value = 1
	Water Source	p-value = 0.53	p-value = 1	p-value = 1	p-value = 1
		95% CI [0.40, 6.42]	95% CI [0.26, 4.54]	95% CI [0.076, 11.10]	95% CI [0.012, 17.45]
	Treating Water	p-value = 0.72	p-value = 1	p-value = 0.36	p-value = 1
		95% CI [0.28, 6.77]	95% CI [0.13, 3.97]	95% CI [0.27, 23.50]	95% CI [0.016, 13.11]
	Water Scarcity	p-value = 1	p-value = 1	p-value = 0.48	p-value = 0.14
		95% CI [0.29, 2.76]	95% CI [0.28, 2.98]	95% CI [0.32, 10.27]	95% CI [0.57, 22.74]
	Toilet Type	p-value = 1	p-value = 0.27	p-value = 1	p-value = 0.034
	Toilet Location	p-value = 1	p-value = 1	p-value = 1	p-value = 1
	Lighting Type	p-value = 0.36	p-value = 0.28	p-value = 0.97	p-value = 0.41
	Cooking Stove Type	p-value = 0.53	p-value = 0.72	p-value = 0.67	p-value = 0.054
	Fuel	p-value = 0.85	p-value = 0.37	p-value = 0.82	p-value = 0.037
