# Supplementary material for: Do monetary incentives encourage local communities to collect and upload mosquito sound data using smartphones? A case study in the Democratic Republic of the Congo
Source: PLoS One. 2025 Aug 7;20(8):e0314122. doi: 10.1371/journal.pone.0314122 (PMC12331038; doi:10.1371/journal.pone.0314122)
Supplement: Attachment — Submitted filename: PONE-D-24-49851_R1_Annotated.pdf [file pone.0314122.s005.pdf]

PLOS ONE

Do monetary incentives encourage local communities to collect and upload mosquito sound data using smartphones? A case study in the Democratic Republic of the Congo

--Manuscript Draft--

**Manuscript Number:**	PONE-D-24-49851R1
**Article Type:**	Research Article
**Full Title:**	Do monetary incentives encourage local communities to collect and upload mosquito sound data using smartphones? A case study in the Democratic Republic of the Congo
**Short Title:**	Incentives for citizen science data collection in the DRC
**Corresponding Author:**	Kieran Storer<br>University of Oxford<br>Oxford, UNITED KINGDOM OF GREAT BRITAIN AND NORTHERN IRELAND
**Keywords:**	Citizen science; Malaria; Ecological Monitoring; public health
**Abstract:**	Malaria is one of the deadliest vector borne diseases affecting sub-Saharan Africa. A suite of systems are being used to monitor and manage malaria risk and disease incidence, with an increasing focus on technological interventions that allow private citizens to remotely record and upload data. However, data collected by citizen scientists must be standardised and consistent if it is to be used for scientific analysis. Studies that aim to improve data collection quality and quantity have often included incentivisation, providing citizen scientists with monetary or other benefits for their participation in data collection. We tested whether monetary incentives enhance participation and data collection in a study trialling an acoustic mosquito sensor. Working with the community in two health districts in the Democratic Republic of Congo, we measured data collection participation, completeness, and community responses. Our results showed mixed responses to the incentive, with more participants interested in the social status and monetary value of the technology used than the monetary incentive itself. The effect of incentives on data collection varied over the course of the trial, increasing participation in the start of the trial but with no effect in the latter half of the trial. Feedback from participants showed that opinions on technology, research objectives, and incentives varied between communities, and was associated with differences in data collection quantity and quality, suggesting that differences in community interest in data collection and the incentives may be more important than the incentive value itself. These results suggest that though there is an initial benefit, extrinsic motivations do not override differences in intrinsic motivations over time, and enhanced communication and dialogue with participants may improve citizen science participation and attitudes.
**Order of Authors:**	Kieran Elisabeth Storer
	Jane P Messina
	Eva Herreros-Moya
	Emery Metelo
	Josué Zanga
	Nono M Muvamba
	Soleil Muzinga
	Rinita Dam
	Marianne Sinka
	Ivan Kiskin
	Josh Everett
	Yunping Li
	Stephen Roberts

Powered by Editorial Manager® and ProduXion Manager® from Aries Systems Corporation

	Kathy Willis
**Response to Reviewers:**	Thank you for the opportunity to submit a revised draft of our manuscript titled "What incentives encourage local communities to collect and upload mosquito sound data using smartphones? A case study in the Democratic Republic of the Congo" to PLOS One. We appreciate the time you and the reviewers have taken to provide valuable feedback and insightful comments. We have made changes to incorporate most of the suggestions provided by reviewers and they are highlighted through the manuscript. Please find below the point-by-point responses and edits made to the paper in line with feedback.<br><br>Journal Requirements:<br>Comment 1: Please ensure that your manuscript meets PLOS ONE's style requirements, including those for file naming.<br>Response: The manuscript has been amended to address the style and naming requirements.<br><br>Comment 2: Please note that funding information should not appear in any section or other areas of your manuscript. We will only publish funding information present in the Funding Statement section of the online submission form. Please remove any funding-related text from the manuscript.<br>Response: The manuscript has been amended to remove funding information from the manuscript.<br><br>Comment 3: When completing the data availability statement of the submission form, you indicated that you will make your data available on acceptance. We strongly recommend all authors decide on a data sharing plan before acceptance, as the process can be lengthy and hold up publication timelines. Please note that, though access restrictions are acceptable now, your entire data will need to be made freely accessible if your manuscript is accepted for publication. This policy applies to all data except where public deposition would breach compliance with the protocol approved by your research ethics board. If you are unable to adhere to our open data policy, please kindly revise your statement to explain your reasoning and we will seek the editor's input on an exemption. Please be assured that, once you have provided your new statement, the assessment of your exemption will not hold up the peer review process.<br>Response: All the raw data from our study is now publicly available at 10.6084/m9.figshare.27332124<br><br>Comment 4: We note that Figure 2 in your submission contain map/satellite images which may be copyrighted. All PLOS content is published under the Creative Commons Attribution License (CC BY 4.0), which means that the manuscript, images, and Supporting Information files will be freely available online, and any third party is permitted to access, download, copy, distribute, and use these materials in any way, even commercially, with proper attribution. For these reasons, we cannot publish previously copyrighted maps or satellite images created using proprietary data, such as Google software (Google Maps, Street View, and Earth). For more information, see our copyright guidelines: http://journals.plos.org/plosone/s/licenses-and-copyright.<br>Response: To comply with the rules regarding CC BY 4.0 the original data showing the health districts of the DRC from the Humanitarian Data Exchange (DRC health Data) was removed and replaced using Creative Commons Attribution Licensed data. The Democratic Republic of the Congo (DRC) GIS data from simplemaps, the GRID3 COD - Health Areas v4.0: Kwilu health areas (GeoPackage), and the GRID3 COD - Health Areas v4.0: Kinshasa health area (GeoPackage) are all accessible under existing CC BY 4.0 licenses. This change in data used in the map of the DRC has also been reflected in the addition of two references for the GeoPackages used, along with the statement of origin in the caption on Figure 2:<br>Center for International Earth Science Information Network (CIESIN), Columbia University, Ministère de la Santé Publique, Hygiène et Prévention, Democratic Republic of the Congo, and GRID3. (2025). GRID3 COD - Health Areas v4.0: Kinshasa health areas (GeoPackage). New York: Columbia University. https://data.humdata.org/dataset/grid3-cod-health-areas-v4-0. Accessed April 3, 2025.<br>Center for International Earth Science Information Network (CIESIN), Columbia University, Ministère de la Santé Publique, Hygiène et Prévention, Democratic Republic of the Congo, and GRID3. (2025). GRID3 COD - Health Areas v4.0: Kwilu

Powered by Editorial Manager® and ProduXion Manager® from Aries Systems Corporation

health areas (GeoPackage). New York: Columbia University. https://data.humdata.org/dataset/grid3-cod-health-areas-v4-0. Accessed April 3, 2025.

Comment 5: We note that Figure 3 and 4 includes an image of a participant in the study.
Response: These images have been removed from the manuscript.

Comment 6. Please review your reference list to ensure that it is complete and correct. If you have cited papers that have been retracted, please include the rationale for doing so in the manuscript text or remove these references and replace them with relevant current references. Any changes to the reference list should be mentioned in the rebuttal letter that accompanies your revised manuscript. If you need to cite a retracted article, indicate the article's retracted status in the References list and also include a citation and full reference for the retraction notice.
Response: The citation list has been reviewed and there were no instances of retracted papers. The reference list was amended with the GeoPackage references for the Kwilu and Kinshasa health districts (shown above), and the references were renumbered to reflect these additions.

Comments from Reviewer 1
Major comments
Comment 1: Lines 79-85: the introduction references the Tanzania study but does not clearly position this manuscript as a follow-up. It could strengthen the manuscript to explicitly state how this work builds on or how it differs from the Tanzania findings. It is recommended that the authors add a sentence explicitly linking the gaps identified in the Tanzania study (for instance, rural-only context) to the objectives of this work.
Response: This study is named directly as a second trial of the mosquito detection application with monetary incentives in line 187. Verbiage specifying the research's focus on the use of monetary extrinsic factors, and how the study builds on the study in Tanzania has been added in lines 205-206, stating: 'This study builds upon our previous work in Tanzania to fill gaps in understanding how monetary incentives alone influence the consistency and quality of audio data collection.'

Comment 2: Regarding the Focus Group Insights, lines 247-253, the discussion of Focus Group Discussions (FGD) results is useful but could benefit from additional depth. For example, higher persistence in Bandundu could be contextualized using participant quotes. A suggestion would be that the authors add representative quotes from FGDs to illustrate key themes and link them to the observed participation trends.
Response: Due to the lack of significant differences in pre-trial FGD responses between Kinshasa and Bandundu it does not feel appropriate to use a FGD response in this section of the results to highlight differences in the groups that were seen later in the trial (such as in the post-trial FGDs). Instead, a quote from a participant listing multiple motivation factors has been added in lines 373-377 to demonstrate how complex participant motivations were prior to trial participation.

Comment 3: The study's inclusion of Bandundu (rural) and Kinshasa (urban) appear to be an opportunity to explore how rural-urban differences influence participation. However, the manuscript does not fully address these dynamics or their potential implications. In lines 401-402, the authors note that differences in participation are not attributable to demographic factors but do not explore other potential influences, such as rural-urban dynamics that may be marked in the two setting of the study. This section could be expanded on to discuss e.g. infrastructure, community cohesion, and leadership differences between Bandundu and Kinshasa.
Response: This study was not aimed at specifically addressing urban versus rural factors for responses to incentivization, and the Kinshasa study sites were not located in the city but in rural parts of the health district (referred to as rural Kinshasa when first introduced in the manuscript, line 188). However, we acknowledge the potential role of proximity to urban areas and have now addressed this in the manuscript discussion lines 547-549, reading: 'These differences may be related to the proximity of the rural Kinshasa sites with the city of Kinshasa, though the access of participants to urban environments and differences in community cohesion were not specifically accounted for in our surveys.'

Minor comments:

Comment 4: Line 46: replace "effecting sub-Saharan Africa" with "affecting sub-Saharan Africa."
Response: This has now been amended in the manuscript.

Comment 5: Discussion: the authors acknowledge that the findings are context-specific but do not discuss how they might be generalised to other settings. The paper could benefit from expanding the discussion to include how differences in infrastructure, culture, or community norms might affect the scalability of the study's approach. Ethical considerations, such as phone retention, may also be under-discussed in terms of their potential impact on the findings or on the design of the study.
Response: To address the generalizability of this research we added a section in lines 702-714 stating: "Our findings show that mobile applications using hardware on budget smartphones can generate useful data with limited interventions and suggests that mobile application development should be more widely considered in citizen science data collection efforts. Future research on the role of incentivization for data collection that utilizes technology should consider how to further disambiguate the inherent benefits in participation related to technological access, such as social status or monetary benefit from retaining equipment. We suggest that with increased mobile phone ownership, this complexity may be overcome by utilizing current phone owners, rather than providing participants with hardware."
The focus group discussions did not include specific questions that would allow us to analyse differences in culture and community norms beyond the motivations participants stated before the trial or their experiences afterwards. Differences in attitude and community norms were addressed in the discussion lines 575-678, and we have added in lines 709-712 a call for more crossectional work that may generate the data necessary to analyse the effects of infrastructure and community norms.

Comment 6: Future Research Directions: the conclusion could benefit from a brief discussion of future research priorities, such as testing alternative incentive models, evaluating long-term engagement strategies, and/or assessing the usability of the acoustic mosquito sensors in the study context.
Response: Future research directions we have added a section to the discussion in lines 702-713: "Future research on the role of incentivization for data collection that utilizes technology should consider how to further disambiguate the inherent benefits in participation related to technological access, such as social status or monetary benefit from retaining equipment. We suggest that with increased mobile phone ownership, this complexity may be overcome by utilizing current phone owners, rather than providing participants with hardware. Additionally, as mobile phone use becomes more popular, opportunities for large scale studies will support more complex statistical approaches to account for the complex structures that underpin intrinsic motivators and responses to extrinsic motivators, such as proximity to urban areas which are potentially obscured by the inferential statistical methods used in this study."

Comment 7: Methodology improvement: the supplementary file provides detailed documentation of participant demographics and focus group questions, which supports the reproducibility of the study. Nevertheless, the modified random walk technique used for participant selection is not sufficiently explained in either the manuscript or the supplementary materials. While the inclusion of demographic comparisons and FGD protocols is quite valuable, the lack of detail about the recruitment method limits the transparency of the methodology. For instance, how were starting points for recruitment selected? Were any randomisation procedures applied to maintain the sampling unbiased? Additionally, minor gaps in the description of the design of text message reminders could also limit reproducibility. It is recommended that the authors provide additional detail or supplementary materials for these two aspects.
Response: Details for how random walks were carried out is included in the methodology in lines 247-249, with details on reducing bias added in line 248: "To reduce the bias of entry point selection, each household was approached sequentially along the street and asked if they wanted to participate, until the number of households required to participate at the site (n=37) was met."
Regarding the methodological details of the SMS reminders, those reminders were not a component of this study but were part of the first study in Tanzania.

Comment 8: Data availability seems to be in compliance with PLOS guidelines. Still, it is important to confirm that the repository cited by the authors includes raw data points

Powered by Editorial Manager® and ProduXion Manager® from Aries Systems Corporation

	(such as the individual participation records and anonymised FGD transcripts), as I was not able to access it. The statistical methods used appear to be appropriate, but I would recommend consulting someone with higher expertise in this matter. The methodology is sufficiently detailed overall. However, minor gaps in the description of participant recruitment (e.g., modified random walk technique) and the design of text message reminders could be further improved.<br>Response: The additional raw data is available now at 10.6084/m9.figshare.27332124.<br><br>Comments from Reviewer 2<br>This study has addressed an important question about the influence of monetary incentives on participation (data collection) in citizen research. The methodology and results have been excellently presented and discussed. The researchers have done an excellent job of using quantitative and qualitative methods to address their study questions. The manuscript is indeed of very high quality.<br>Here are a couple of comments that I recommend that the authors consider:<br>Comment 1: The topic of the study is not congruent with the study objective or question. The title “What incentives encourage local communities to collect and upload mosquito sound data by using smartphones? A case study in the Democratic Republic of the Congo” suggests that the study has evaluated different type of incentive while it has specifically assessed the influence of “monetary incentives” A title that would fit better with the study objective/question/scope could be “Do monetary incentives encourage local communities to collect and upload mosquito sound data by using smartphones? A case study in the Democratic Republic of the Congo”<br>Response: The title of the manuscript has been adjusted to account for this suggestion.<br><br>Comment 2: The researchers may have made efforts to adjust for and discuss confounding inherent on the research design but need to describe this more clearly in the report. Also, I recommend that authors highlight known limitations of the main inferential statistical approach used in comparative analysis.<br>Response: A sentence has been added in the methods to address the use of post-hoc test to reduce risk of type 1 error in line 341.<br>To address the limitation of inferential statistics, a section has been added to the discussion in lines 709-712, suggesting future studies use cross-sectional data that may allow for more complex modelling approaches in analysis.
**Additional Information:**	
**Question**	**Response**
**Financial Disclosure**<br><br>Enter a financial disclosure statement that describes the sources of funding for the work included in this submission. Review the submission guidelines for detailed requirements. View published research articles from *PLOS ONE* for specific examples.<br><br>This statement is required for submission and **will appear in the published article** if the submission is accepted. Please make sure it is accurate.	Yes

**Funded studies**<br>Enter a statement with the following details:<br>• Initials of the authors who received each award<br>• Grant numbers awarded to each author<br>• The full name of each funder<br>• URL of each funder website<br>• Did the sponsors or funders play any role in the study design, data collection and analysis, decision to publish, or preparation of the manuscript?<br><br>Did you receive funding for this work?	
Please add funding details.<br>as follow-up to "**Financial Disclosure**<br><br>Enter a financial disclosure statement that describes the sources of funding for the work included in this submission. Review the submission guidelines for detailed requirements. View published research articles from *PLOS ONE* for specific examples.<br><br>This statement is required for submission and **will appear in the published article** if the submission is accepted. Please make sure it is accurate.<br><br>**Funded studies**<br>Enter a statement with the following details:<br>• Initials of the authors who received each award<br>• Grant numbers awarded to each author<br>• The full name of each funder<br>• URL of each funder website<br>• Did the sponsors or funders play any role in the study design, data collection and analysis, decision to publish, or preparation of the manuscript?<br><br>Did you receive funding for this work?"	KES, JM, EHM, EM, JZ, SM, NMM, RD, MS, IK, JE, YL, SR, and KJW were funded by the Bill and Melinda Gates Foundation Grant Award Number OPP1209888.<br><br>https://www.gatesfoundation.org/<br><br>The Bill and Melinda gates foundation had no role in study design, data collection or analysis, decision to publish, or preparation of the manuscript.
Please select the country of your main research funder (please select carefully as in some cases this is used in fee calculation).	UNITED STATES - US

as follow-up to "**Financial Disclosure**<br><br>Enter a financial disclosure statement that describes the sources of funding for the work included in this submission. Review the submission guidelines for detailed requirements. View published research articles from *PLOS ONE* for specific examples.<br><br>This statement is required for submission and **will appear in the published article** if the submission is accepted. Please make sure it is accurate.<br><br>**Funded studies**<br>Enter a statement with the following details:<br>• Initials of the authors who received each award<br>• Grant numbers awarded to each author<br>• The full name of each funder<br>• URL of each funder website<br>• Did the sponsors or funders play any role in the study design, data collection and analysis, decision to publish, or preparation of the manuscript?<br><br>Did you receive funding for this work?"	
**Competing Interests**<br><br>Use the instructions below to enter a competing interest statement for this submission. On behalf of all authors, disclose any competing interests that could be perceived to bias this work—acknowledging all financial support and any other relevant financial or non-financial competing interests.<br><br>This statement is required for submission and **will appear in the published article** if the submission is accepted. Please make sure it is accurate and that any funding sources listed in your Funding Information later in the submission form are also declared in your Financial Disclosure statement.	The authors have declared that no competing interests exist.

View published research articles from *PLOS ONE* for specific examples.<br><br>**NO authors have competing interests**<br><br>Enter: *The authors have declared that no competing interests exist.*<br><br>**Authors with competing interests**<br><br>Enter competing interest details beginning with this statement:<br><br>*I have read the journal's policy and the authors of this manuscript have the following competing interests: [insert competing interests here]*<br><br>* typeset	
**Ethics Statement**<br><br>Enter an ethics statement for this submission. This statement is required if the study involved:<br><br>• Human participants<br>• Human specimens or tissue<br>• Vertebrate animals or cephalopods<br>• Vertebrate embryos or tissues<br>• Field research<br><br>Write "N/A" if the submission does not require an ethics statement.<br><br>General guidance is provided below. Consult the submission guidelines for detailed instructions. **Make sure that all information entered here is included in the Methods section of the manuscript.**	The University of Oxford have sponsored the study. This study was reviewed and approved by the Oxford Tropical Research Ethics Committee (OxTREC Reference 548-19) and approved by the Kinshasa School of Public Health (ESP/CE/91B/2021). Informed written consent was obtained from all interviewees prior to the focus group discussions and the quantitative feedback survey. Consent forms signed by all the participants included the release of summary findings and details of individual responses from this study. Potential participants were informed of the voluntary nature of the study and had at least 24 hours to consider taking part.

Powered by Editorial Manager® and ProduXion Manager® from Aries Systems Corporation

**Format for specific study types**<br><br>**Human Subject Research (involving human participants and/or tissue)**<br>• Give the name of the institutional review board or ethics committee that approved the study<br>• Include the approval number and/or a statement indicating approval of this research<br>• Indicate the form of consent obtained (written/oral) or the reason that consent was not obtained (e.g. the data were analyzed anonymously)<br><br>**Animal Research (involving vertebrate animals, embryos or tissues)**<br>• Provide the name of the Institutional Animal Care and Use Committee (IACUC) or other relevant ethics board that reviewed the study protocol, and indicate whether they approved this research or granted a formal waiver of ethical approval<br>• Include an approval number if one was obtained<br>• If the study involved *non-human primates*, add *additional details* about animal welfare and steps taken to ameliorate suffering<br>• If anesthesia, euthanasia, or any kind of animal sacrifice is part of the study, include briefly which substances and/or methods were applied<br><br>**Field Research**<br><br>Include the following details if this study involves the collection of plant, animal, or other materials from a natural setting:<br>• Field permit number<br>• Name of the institution or relevant body that granted permission	
**Data Availability**<br><br>Authors are required to make all data underlying the findings described fully available, without restriction, and from the time of publication. PLOS allows rare exceptions to address legal and ethical concerns. See the PLOS Data Policy and FAQ for detailed information.	Yes - all data are fully available without restriction

Powered by Editorial Manager® and ProduXion Manager® from Aries Systems Corporation

A Data Availability Statement describing where the data can be found is required at submission. Your answers to this question constitute the Data Availability Statement and **will be published in the article**, if accepted.<br><br>**Important:** Stating 'data available on request from the author' is not sufficient. If your data are only available upon request, select 'No' for the first question and explain your exceptional situation in the text box.<br><br>Do the authors confirm that all data underlying the findings described in their manuscript are fully available without restriction?	
**Describe where the data may be found in full sentences. If you are copying our sample text, replace any instances of XXX with the appropriate details.**<br><br>• If the data are **held or will be held in a public repository**, include URLs, accession numbers or DOIs. If this information will only be available after acceptance, indicate this by ticking the box below. For example: *All XXX files are available from the XXX database (accession number(s) XXX, XXX.*).<br>• If the data are all contained **within the manuscript and/or Supporting Information files**, enter the following: *All relevant data are within the manuscript and its Supporting Information files.*<br>• If neither of these applies but you are able to provide **details of access elsewhere**, with or without limitations, please do so. For example:<br><br>*Data cannot be shared publicly because of [XXX]. Data are available from the XXX Institutional Data Access / Ethics Committee (contact via XXX) for researchers who meet the criteria for access to confidential data.*<br><br>*The data underlying the results presented in the study are available from (include the name of the third party*	Data and analysis files are available from figshare (10.6084/m9.figshare.27332124).

and contact information or URL).<br>• This text is appropriate if the data are owned by a third party and authors do not have permission to share the data.<br><br>* typeset	
Additional data availability information:	

Manuscript Click here to access/download;Manuscript;Manuscript.docx

Do monetary incentives encourage local communities to collect and upload mosquito sound data using smartphones? A case study in the Democratic Republic of the Congo

Short Title: Incentives for citizen science data collection in the DRC

Kieran E. Storer[1*], Jane P. Messina[2], Eva Herreros-Moya[1], Emery Metelo[3,4¶], Josué Zanga[4¶], Nono M. Mvuama[4¶], Soleil Muzinga[4¶], Rinita Dam[5&], Marianne Sinka[1&], Ivan Kiskin[6&], Josh Everett[1&], Yunpeng Li[7&], Stephen Roberts[8&], and Katherine J. Willis[1]

Affiliations

[1] Department of Biology, University of Oxford, Oxford, United Kingdom

[2] School of Geography and the Environment, Oxford University Centre for the Environment, University of Oxford, Oxford, United Kingdom

[3] Department of Entomology, National Institute for Biomedical Research, Kinshasa, Democratic Republic of the Congo

[4] Kinshasa School of Public Health, University of Kinshasa, Kinshasa, Democratic Republic of the Congo

[5] Warwick Medical School, University of Warwick, Coventry, United Kingdom

[6] Surrey Institute for People-Centred AI, Centre for Vision Speech and Signal Processing, University of Surrey, Guildford, United Kingdom

[7] Centre for Oral, Clinical, and Translational Sciences, King’s College London, London, United Kingdom

[8] Department of Engineering Science, University of Oxford, Oxford, United Kingdom

*Corresponding Author

E-mail: kieran.storer@biology.ox.ac.uk (KES)

[¶] EM, JZ, NMM, and SM contributed equally to this work

[&] RD, MS, IK, JE, YL, and SR also contributed equally to this work

Attribution

KES contributed to data curation, formal analysis, methodology, visualization, and writing the original draft. JPM contributed to formal analysis and visualization. EHM contributed to conceptualization, investigation, methodology, project administration, supervision, visualization, and the review and editing of the manuscript. EM, JZ, NMM, and SM contributed to conceptualization, investigation, methodology, data collection, and review and editing of the manuscript. RD, MS, IK, JE, YL, and SR contributed to conceptualization, methodology, and review and editing of the manuscript. KJW contributed to funding acquisition, methodology, supervision, and the review and editing of the manuscript.

Abstract

Malaria is one of the deadliest vector borne diseases affecting sub-Saharan Africa. A suite of systems are being used to monitor and manage malaria risk and disease incidence, with an increasing focus on technological interventions that allow private citizens to remotely record and upload data. However, data collected by citizen scientists must be standardised and consistent if it is to be used for scientific analysis. Studies that aim to improve data collection quality and quantity have often included incentivisation, providing citizen scientists with monetary or other benefits for their participation in data collection. We tested whether monetary incentives enhance participation and data collection in a

study trialling an acoustic mosquito sensor. Working with the community in two health districts in the Democratic Republic of Congo, we measured data collection participation, completeness, and community responses. Our results showed mixed responses to the incentive, with more participants interested in the social status and monetary value of the technology used than the monetary incentive itself. The effect of incentives on data collection varied over the course of the trial, increasing participation in the start of the trial but with no effect in the latter half of the trial. Feedback from participants showed that opinions on technology, research objectives, and incentives varied between communities, and was associated with differences in data collection quantity and quality, suggesting that differences in community interest in data collection and the incentives may be more important than the incentive value itself. These results suggest that though there is an initial benefit, extrinsic motivations do not override differences in intrinsic motivations over time, and enhanced communication and dialogue with participants may improve citizen science participation and attitudes.

Introduction

Mobile phone usage in sub-Saharan Africa reached 489 million users in 2023, with smartphones making up 51% of mobile phones due to improvements in mobile phone access and network connections [1–3]. With increasing smartphone ownership, their use for citizen science data collection has also increased in tandem, making use of the built-in camera, sensors, and GPS to generate and record data [4–7]. Citizen science projects have ranged widely, and have been increasingly used in healthcare settings, including for monitoring diseases such as malaria by tracking outbreaks, monitoring drug stocks, reminding patients of medical appointments, and recording mosquito larval habitats and populations [for examples see: 8–15]. As the amount and type of data collected by private citizens increases there are opportunities for developing high-resolution data repositories that widen citizen participation to include a greater range of demographic groups.

Previous research on citizen science data collection has shown that gathering data for research or healthcare purposes can help to improve educational outcomes [16], make data collectors feel more connected to their community [17], and improve health outcomes [18]. But how do researchers motivate people to participate and collect high-quality data? Firstly, there is need to understand the roles that intrinsic and extrinsic motivations play in citizen scientist participation and data collection quality. Intrinsic motivation factors are 'people's spontaneous tendencies to be curious and interested, to seek out challenges and to exercise and develop their skills and knowledge, even in the absence of operationally separable rewards' [19]. Intrinsic motivation is influenced by local and regional cultures, education, and familial values [20]. In contrast, extrinsic motivators are 'behaviours done for reasons other than their inherent satisfactions' [21], such as monetary gain [22]. Efforts to improve data collection typically target these different motivation factors. Studies have shown that monetary incentives can be used in health care and ecological monitoring schemes to improve participation [23–27] and that intrinsic factors can be harnessed to improve data collection participation and quality by improving learning opportunities and using participant knowledge [28–31].

The complex spatiotemporal variability inherent to motivation factors [32,33] and the theoretical potential for a trade-off between them [21] make understanding their influence essential to improving data collection quality and scope while optimising resource allocation. To study participants' motivations and assess the functionality of incentive applications to promote community involvement in the collection of high-quality long-term biological data, we previously ran a data collection trial using incentives and SMS text reminders for collecting mosquito audio data in Tanzania using the HumBug tool [34]. This tool is a smartphone audio sensor combined with a modified bed net designed to temporarily trap mosquitoes and guide them towards a budget smartphone running the MozzWear app that records the flight tone of host seeking mosquitoes overnight to determine the abundance and

diversity of mosquitoes present using their sound [35] (Fig. 1).; For details on the Humbug tool, also see [36–42].

Fig. 1: HumBug Tool configuration, showing the data collection, upload, and analysis pipeline (reproduced from Sinka et al., 2021).

The smartphone app MozzWear used in the HumBug tool records mosquito flight tone data overnight and provides a secure connection to a server at the University of Oxford where the data is uploaded via the mobile or Wi-Fi data network. The data then runs through an algorithm pipeline that first detects the mosquito flight tone from background noise and then identifies the mosquito species using their acoustic signature [35–37,40]. As such, the HumBug tool is a good example of using smartphone technologies to provide an accessible biological data collection methodology to identify and monitor vectors of human disease - in this case, mosquitoes.

Previous work in Tanzania showed that providing homeowners with monetary incentives and SMS reminders to record and upload mosquito data did not significantly increase the number of uploads when compared to the control group [34]. Instead, the study found that other factors influenced data collection efforts. However, it did not examine in detail what these other factors might be. To better understand, therefore, how a monetary extrinsic factor may affect data collection practices in the use of the HumBug tool, we ran a second data collection trial in a different community within two districts of the Democratic Republic of the Congo: Bandundu, and rural Kinshasa. In this study we aimed to test whether monetary extrinsic incentives encouraged: i) participant data collection activity during the trial period; ii) participant effort (the number of uploads per participant made during the sampling period, indicative of following trial protocols); and iii) the persistence of participation over time (whether trial participants continued to upload data throughout the sampling period). To address these questions, we compared participant activity (weeks active) and sampling effort (number of uploads)

over the sampling period to assess differences in participation associated with receiving incentives for data collection. This study builds upon our previous work in Tanzania to fill gaps in understanding how monetary incentives alone influence the consistency and quality of audio data collection.

Methods

The study ran from April to November of 2022 in two districts of the Democratic Republic of the Congo (DRC): rural Kinshasa, and Bandundu. Our collaborators at the University of Kinshasa and the University of Bandundu selected participants, conducted demographic surveys and pre- and post-trial focus group discussions, and ran the study. Data analysis and writing of the manuscript were carried out by the University of Oxford team. All leaders of communities, health zone officials, and participants provided signed consent forms for the study, and the study was approved by the University of Oxford Tropical Research Ethics Committee and the University of Kinshasa Public Health Ethics Committee. Informed written consent was obtained from all interviewees prior to the focus group discussions and the quantitative feedback survey. Consent forms signed by all the participants included the release of summary findings and details of individual responses from this study. Potential participants were informed of the voluntary nature of the study and had at least 24 hours to consider taking part. Efforts were made to create a safe place for sharing experiences during the focus group discussions.

Study Locations

Geographically, the DRC covers an area of approximately 2,345,409 km^2. The country is dominated by the Congo River basin surrounded by high plateaus, resulting in high precipitation and thick tropical forest within the basin and grassland in the plateaus above. In the DRC, malaria is a major cause of

illness and death. Worldwide, 12% of the total malaria cases occurred in the DRC in 2022, causing 60% of hospital visits in the country [43,44].

Kinshasa is the capital city of the DRC and the largest city in central Africa. With ~16 million population it is the third largest mega-city on the continent. The Bandundu district has a total of 143,435 people and covers an area of 222 Km² (Figure 2). Participants in our research were from the Bu and Mikondo health districts of Kinshasa, and the Trois Rivières and Caravane health districts of Bandundu.

Fig. 2: Map of the Democratic Republic of Congo, modified from simplemaps.com [45] and GRID3 COD - Health Areas v4.0 [46,47] *under CC BY 4.0 license, showing the location of study districts; Bandundu in red and Kinshasa in blue. On the zoom inset, black points show the location of study sites. Mapping was carried out using QGIS 3.30.0 [48].*

Each district in our study had two trial sites: a control site and an incentive site. The selection of participants from our four trial sites took place from April 2 to June 30, 2022. In each health district, authorities of the different Health Zones, the village chiefs, and heads of districts were contacted to gain permission for the study and to involve local leadership. Meetings with local leaders were carried out to plan the recruitment of households for the study, and to allocate which health districts would be the control treatment (Trois Rivières in Bandundu and Bu in Kinshasa) and the incentive treatment (Caravane in Bandundu and Mikondo in Kinshasa). These health districts will hereafter be referred to as a trial sites for clarity.

Households were recruited using a modified random walk technique from an entry point chosen from the main entrances to the village/street selected by local health district officials. To reduce the bias of entry point selection, each household was approached sequentially along the street and asked if they

wanted to participate, until the number of households required to participate at the site (n=37) was met. Trial participants were admissible if they were 18+ years old, owned or had access to a personal mobile phone, were residents in Bandundu/Kinshasa throughout the study, and were willing and capable of providing a signed consent form.

Ahead of the trial, training of the interviewers and moderators took place over a day in each of the trial sites to teach local health officials and local leadership how to run interviews and support participants in the trial. Demonstrators were also taught how to install and use the HumBug tool and MozzWear App and were given a presentation of the trial objectives. Finally, interviewers were trained on how to administer the demographic questionnaires to participants who agreed to take part in the trial (DHS Phase 8 Questionnaire, S1 Appendix).

Moderators ran pre-trial focus group discussions (FGDs) to confirm that participants understood the purpose of the study, how the study was designed, and how to operate the HumBug tool and MozzWear application. The pre-trial FGDs were also used to discuss and understand the challenges that participants may face, their motivations for taking part in the study, and what they thought the impact would be on their lives. The pre- and post- trial FGD questions can be found in the S2 Appendix. The reasons participants gave for joining the study were categorized into common themes of access to electricity, financial incentive, use of a phone, provision of bed nets, contributing to health improvements and malaria control, or gaining personal knowledge. Participant FGDs responses were then structurally coded for analysis [49].

Control group participants were provided with the HumBug tool (a smartphone running the MozzWear app and the HumBug bed net), and one dollar to pay for an internet connection. The incentive group was provided with the HumBug tool and one dollar for an internet connection, and an additional ten

dollars each month paid via airtime to their mobile phones. Participants were instructed to place the smartphone with the MozzWear app in the HumBug net and start the record function at 18:00 hrs and turn it off at 06:00 hrs on their allocated weekly recording day during the trial period (16 weeks total). Recordings were split at one-hour intervals automatically in the MozzWear app to prepare recordings for algorithmic analysis. As such a complete recording effort would show 12 recordings. The recordings were then uploaded by participants to the remote server when they connected to the internet. Once participants were taught how to use the Humbug tool there was no contact between the research team and the participants.

The research team conducted post-trial FGDs to understand the participants' experience of using the HumBug tool and participating in the study. Questions included whether they liked using the MozzWear app, whether they liked using the HumBug net, how the trial personally affected them, what they would want to be different about the trial in the future, whether they would participate in the future, and if they received an incentive whether it was enough money. Data from the post-trial FGDs was coded for analysis by themes of response to questions as described for the pre-trial FGDs.

Data Analysis

To compare the effect of providing incentives to the participants to upload mosquito audio data, the number of active participants (participants who uploaded any data) and the number of uploads were counted during each trial week. Participant identification numbers were used to assess the upload counts and weekly activity by location (Kinshasa or Bandundu districts) and experimental group (control or incentive). Counts of uploads, weeks of participation, and the average number of participants each week were compared using Wilcoxon Rank Sum tests [50] and Kruskal-Wallis tests [51] with a post-hoc Dunn test [52] to assess if there was a significant difference in participation between the experimental groups/locations over the study period and reduce potential type 1 error

associated with multiple comparisons. The number of participant uploads was broken down for each week of the trial to assess whether incentives improved data collection persistence throughout the study and was also compared using Wilcoxon Rank Sum Tests. Data on income, sex, age, education, and profession were also evaluated as explanatory variables to explain variation in data collection between groups. Comparisons of the demographic data of each district, trial group, and combination of the two, were made using Wilcoxon Rank Sum tests and Chi-squared tests. The pretrial FGD data was analysed using Wilcoxon Rank Sum tests to compare differences in pre-trial motivations for participation. The post-trial FGD data was analysed using a Fisher's Exact Test to assess the correlation between district and trial groups on attendance for the post-trial FGD and whether participants would take part in a future trial, and phone return. All plots were produced using ggplot2 [53].

Data management

Personal data generated in the form of signed consent forms, personal mobile phone numbers, interviews, and/or focus group audio recordings, were stored following the 2018 Data Protection Act on a secure administrative database on a University of Oxford server.

Results

Pre-Trial Focus Group Discussions

Pre-trial focus group discussions (FGDs) were run in three groups of 12-13 participants in each trial site (37 people per trial site, 148 participants in total). In the pre-trial FGDs, themes identified for participant motivation were monetary benefit (63.5% of all participants), contributing to science/health (33.1%), having a phone (29.7%), electricity access (27.0%) (not a component of this study), the HumBug net (6.7%), and gaining personal knowledge (5.4%). Participants were typically motivated by multiple factors. As a participant in Bandundu describes ‘To do the job well, I have to be motivated,

in the sense that I have to have the material for the job first, and then I have to be paid a good payment so that I can do a good job". There was no significant difference in the number of motives each participant listed at the start of the study between Bandundu and Kinshasa ($p > 0.05$).

Statistical comparisons using Wilcoxon Rank Sum Tests showed that trial site participants in Kinshasa mentioned electricity significantly more as a reason to participate in the trial compared to Bandundu trial site participants (W = 3774, p-value < 0.001). Comparing treatment groups across both districts, electricity access was mentioned more frequently in the control groups compared to the incentive groups (W = 2072, p-value < 0.001). Between the treatment groups in Bandundu, electricity was more frequently mentioned by the control group (control n=6, incentive n=0; W = 795.5, p-value = 0.012) and personal knowledge (on topics such as mosquito control and malaria prevention) was more commonly cited by the incentive group (control n=0, incentive n=4; W = 610.5, p-value = 0.042). In Kinshasa, the control group mentioned electricity (control n = 23, incentive n = 11; W = 906.5, p-value = 0.005) and contributing to science/health (control n = 15, incentive n = 6; W = 851, p-value = 0.022) significantly more than the incentive group. There were no other significant differences between districts, trial groups, or the trial groups within districts (p-value>0.05).

Demographic Survey

Demographic survey data were assessed to identify if they contributed to differences in recording ability between districts, treatment groups, and treatment groups within districts. There were significantly more adults in the homes of participants in the Kinshasa district sites compared to the Bandundu district sites (W = 1680, p-value = 0.017). Between the treatment groups in both districts, there were no significant differences in any demographic response variables. Within Kinshasa, there was significantly less water scarcity in the control group (29% experiencing water scarcity), and compared to the incentive group (45% experiencing water scarcity) (odds ratio = 0.09, p-value = 0.021)

and there was a significant difference in the method of home lighting, with significantly more homes in the control group using a rechargeable flashlight, torch, or lantern (p-value = 0.016). Within Bandundu there were significantly more children in the incentive group compared to the control group (W = 675, p-value = 0.024), with one more child per house in the study on average. Additionally, there was a significant difference in toilet types between the Bandundu trial groups (p-value = 0.033). These were the only cases out of 88 comparisons that were significantly different between districts and trial groups (S3 Table).

Comparing Districts and Trial Groups

Some participants decided to withdraw from the trial during its course and others failed to upload data, resulting in overall participation of 33 from the control group and 31 from the incentive group in the Kinshasa districts (n=64), and 33 from the control group and 35 from the incentive group in Bandundu districts (n=68), totalling 132 participants. The withdrawal rate from the study was 7.4% from the control sites and 10.4% from incentive sites across both districts. The reasons cited for formal withdrawal from the trial were lack of monetary benefit, unplanned move from the trial area, and loss of the smartphone. Comparisons in participation between trial districts using Wilcoxon Continuity tests showed that Bandundu participants uploaded data more weeks of the trial period ($W = 1589$, $p < 0.001$) and had more total uploads ($W = 1825.5$, $p = 0.009$), than participants in Kinshasa, shown in Figure 3.

Fig. 3: Comparison of a) the total number of uploads per participant in Bandundu and Kinshasa and b) the number of weeks that each participant was active in Bandundu and Kinshasa. Bandundu is shown in red, and Kinshasa is shown in blue. Boxes show the interquartile range (IQR), with whiskers showing the upper and lower 25% of the data, and points showing the outliers.

Comparing treatment groups across both districts there was no difference in participation between the control group and the incentive group for either total uploads (W = 2409.5, p-value = 0.88), or participation weeks (W = 2510.5, p-value= 0.79). Within Kinshasa there was no significant difference between treatment groups in either uploads (W = 418.5, p-value = 0.057) or weeks of activity (W = 428, p-value = 0.073). In Bandundu, there were significantly more total uploads in the control group compared to the incentive group (W = 845.5, p-value = 0.026) but no significant difference in the average number of active weeks of each participant (W = 777, p-value = 0.13) (Table 1). There were no significant relationships found between in the demographic variables that were captured in the demographic questionnaire survey and the number of uploads or weeks of activity.

*Table 1: Results of the Mann Whitney-U Tests for the trial group comparisons, with * to denote significance, p-value < 0.05 *, p-value<0.01 **.*

**Comparison**		**Response Variable**	**Group**	**Mann-Witney U**	**Median**	**IQR**	**P-Value**
**District**		**Uploads**	**Kinshasa**	W=1825.5	117.5	79.75-161.75	0.009486***
			**Bandundu**		160	107-186	
		**Weeks**	**Kinshasa**	W=1589	11	8-14.25	0.0002879***
			**Bandundu**		15	12-16	
**Treatment**		**Uploads**	**Control**	W=2409.5	136	73-186	0.8822
			**Incentive**		140	94.5-172.5	
		**Weeks**	**Control**	W = 2510.5	13	7-16	0.7852
			**Incentive**		13	10-15	
**Bandundu**	**Treatment**	**Uploads**	**Control**	W=845.5	180	122.25-193.5	0.02643*

			**Incentive**		149.5	91-177.75	
		**Weeks**	**Control**	W=777	16	12-16	0.1307
			**Incentive**		13.5	11.5-16	
**Kinshasa**	**Treatment**	**Uploads**	**Control**	W=418.5	99	62-164	0.05705
			**Incentive**		140	96.5-160	
		**Weeks**	**Control**	W=428	11	6-14	0.07298
			**Incentive**		12	10-14.5	

Activity and Effort over Time

Activity Persistence

The percentage of active participants was catalogued weekly to assess whether the monetary incentive affected persistence in data collection over the trial period (Fig. 4). At the start of the trial, the Kinshasa incentive group had the highest participation (87.1%), followed by the Bandundu incentive (83.3%), Bandundu control (80.5%), and Kinshasa control (70.3%). Participation was approximately stable for the first half of the trial before declining from week 8. At the end of the trial both the Kinshasa control and Kinshasa incentive group had dropped to 30-35% participation, Bandundu incentive dropped to 50% participation, and Bandundu control maintained 77.7% participation.

Fig. 4: The percentage of participants that activated the MozzWear app in each trial group during each week in the trial. The coloured lines coordinate with trial groups, showing Bandundu control in orange, Bandundu incentive in green, Kinshasa control in pink, and Kinshasa incentive in blue. Lines were fitted using locally

estimated scatterplot smoothing (LOESS), with the 95% confidence interval for each treatment group indicated in grey.

Data Collection Effort

Data collection effort over the trial showed that in the first week the Kinshasa incentive group had the highest average uploads per active participant (12.26) followed by the Bandundu control (11.69), Bandundu incentive (10.83), and Kinshasa control (10.54). Data collection effort declined at around week seven, and at the end of the trial, the upload effort dropped for all groups, finishing in descending order with Bandundu control (11.29), Bandundu incentive (10.39), Kinshasa incentive (10.8), and Kinshasa control (9.23). Over the course of the study, Bandundu had significantly more uploads per participant compared to Kinshasa (W = 1825.5, p-value = 0.009), and the Bandundu control group had higher data collection effort than the incentive group (W=845.5, p-value = 0.026). but there was no significant difference between the control and incentive groups overall or within Kinshasa. Figure 5 below shows the weekly comparisons in data collection effort over the course of the trial between the trial districts, treatment types, and treatments within districts. Bandundu had a trend of higher uploads in weeks 2-4, 8, and 11 (p-value < 0.1), and significantly higher uploads in weeks 9, 10, and 12-16 (p-value < 0.05) compared to the Kinshasa groups. Comparisons of the treatment groups showed that the control groups had higher participation than the incentive groups in the final three weeks of the trial (p-value <0.05). Within Bandundu, the control group had higher participation in weeks 14 and 16 of the trial compared to the incentive group (p-value <0.05), and within Kinshasa, the control group had trends of lower participation than the incentive group in weeks 1 and 9 of the trial (p-value < 0.1)

Fig. 5: Week-to-week comparisons of collection effort between the trial districts, and incentive and control groups overall and within Kinshasa and Bandundu. The magnitude of the bars shows the difference in uploads per person from the first group in panel subtitle relative to the second. Non-significant differences are shown in grey; trends are shown in green (p-value < 0.1), significant results in yellow (p-value < 0.05), and highly

significant differences are shown in red (p-value < 0.001). The whiskers around each bar show the standard error.

Post-Trial Focus Group Discussions

The focus group discussions after the trial were attended by 27 participants from Kinshasa and 26 from Bandundu equating to ~35% of the original trial participants. In comparison to the 12 pre-trial FGDs, only 6 post-trial FGDs were run. Post-trial FGD attendance was lower in the Kinshasa incentive group compared to other groups (n = 8, p-value = 0.098, 95% CI: [0.0995, 1.2767] odds ratio: 0.368). All returning participants indicated that they had positive experiences with the HumBug sensor and a positive experience with the HumBug bed nets. When asked about the effect of the trial on their life, a respondent from the Bandundu incentive site said, "I was doing this work without someone to command me (...) it gave me the sense of responsibility."

There was a lower interest in future participation for control groups (n=12) compared to the incentive groups (n=25), and lower interest in future participation in Kinshasa (44.4% of respondents) compared to Bandundu (96.2% of respondents). Both district and trial group were significantly associated with participants interest in future participation (p-value < 0.001, 95% CI: [1.801, 5.468], odds ratio: 3.102), showing that in Bandundu the control group was 10% less likely to say they would participate in the future than the Bandundu incentive group, and in Kinshasa the control group was 60% less likely to participate in the future compared to the Kinshasa incentive group.

When asked about challenges or things they wanted to change in the trial, electricity access was mentioned more frequently in the Kinshasa incentive group (W = 32, p-value = 0.007) and the Bandundu control group (W = 129, p-value = 0.008) compared to the other trial group in their respective districts. Financial compensation/increased compensation was mentioned by 17

respondents, but did not vary significantly between trial groups. When queried on how to improve the study, a respondent indicated a desire for more interaction and information, "When we send the data, on your part, we need a sign to know if the data has arrived or not (...) until the end, we had no communication."

Finally, only participants in the Kinshasa control group mentioned wanting to keep the smartphone (n=12). Despite not being discussed in other post-trial FGDs, phone retention was an issue at the end of the study. In total, there were 33 phones retained by participants across the districts, with a higher likelihood for the Kinshasa incentive group and for the Bandundu control group to retain phones, compared to the other trial group in their district (p-value=0.00516, 95% CI [1.567, 74.361], odds ratio: 9.177). Additionally, nine phones were reported as lost or stolen by the end of the trial period. As one participant phrased it from the control trial group in Kinshasa, “I want to be left with the phones because we will be laughed at (…) if you take them away from us, they will say that we participated in vain.”

Discussion

Our results demonstrate that in the DRC there was no significant difference in the number of audio recordings uploaded or the number of weeks that participants were active between the groups receiving financial incentives and the groups that did not. The previous HumBug study in Tanzania similarly showed no significant effect of incentives (monetary incentives, text message reminders, or their combination) on the total audio recordings uploaded [34]. We found there was no significant effect of demographics such as age, sex, or income on the number of uploads or participation weeks, nor were there significant differences in these demographic traits between the districts, trial groups, or trial groups within districts. The only significant effect on the number of uploads and active weeks was study location which showed that participation was overall higher in Bandundu compared to Kinshasa,

and a difference between the control and treatment group uploads in Bandundu due to a higher recording effort of the control group. This suggests that differences between the districts are not attributable to demographic effects, and instead, there may be other differences, such as intrinsic motivations or community leadership, resulting in higher numbers of uploads in Bandundu compared to the Kinshasa sites. These differences may be related to the proximity of the rural Kinshasa sites with the city of Kinshasa, though the access of participants to urban environments and differences in community cohesion were not specifically accounted for in our surveys.

Our study results also show that incentivisation did not significantly affect audio uploads or activity within Kinshasa and showed that the control group had more audio uploads in Bandundu compared to the incentive group. Previous studies have shown that if incentivised actions align with social norms, incentives can bolster desired behaviours [54], however, incentives can also weaken participants' intrinsic motivations—a phenomenon known as the 'crowding out' effect. This occurs when the anticipated intrinsic benefits of participation, such as the satisfaction of contributing, are diminished by the introduction of external incentives [55]. The lack of response to incentives in our study suggests that incentives were neither supporting intrinsic motivations nor diminishing them. Notably, for logistical reasons this trial used individual health districts as trial groups. Differences in response to incentives may be more strongly attributed to local culture and attitudes than treatment group. In the future, a randomised control trial that assesses participants' existing motivations could more effectively capture variations both among and within localities engaged in data collection. Additionally, further work is needed to understand what the tipping points are for incentives to significantly motivate participation, and what behaviours may either be unaffected by incentivisation or diminished by it.

Although incentives did not affect the overall number of data uploads across districts, there were differences in the persistence of data collection participation and effort. The participation persistence

of the trial groups showed that the Bandundu control group had approximately the same amount of participation from the start to the end of the trial, but that the other three trial groups experienced significant declines in the latter half of the trial. A review paper of citizen science studies found that studies that provided long term training and contact with organisers were the most successful at engaging participants [56]. The declines in robust participation towards the end of the trial in our study may be attributed to the lack of engagement with participants that was necessary to test the usability of the data collection methodology with low intervention. The low contact resulted in several post-trial focus group respondents indicating they would have liked more contact, despite all participants saying the sensor and application were easy to use. Although participants had positive attitudes regarding the data collection and responsibility (supporting results from other studies [57]) access to data and collaborators may further improve participants' feelings about their participation and their contributions [58].

Participant-researcher contact may be why our previous trial in Tanzania found that text reminders, both alone and paired with an incentive, positively affected persistence in data collection, despite the monetary incentive alone not showing a significant effect in that study system [34]. The declines in participation persistence and effort seen in this study support this previous finding that incentives alone are not enough to motivate participation. The tipping point in the middle of the study where participation dropped suggests that participants do not need further encouragement or motivation in the short term to collect Humbug data. In longer term contexts however, consistent contact and communication may be necessary to continue to motivate participants, and we have found that monetary incentives do not accomplish this, and in fact appear to only increase participation in the early phases of the study.

The differences in upload effort between our trial groups did not follow patterns relating to incentivisation. The data collection effort in Bandundu shows that the control group performed similarly to the incentive group for most of the trial, with the control group performing significantly better in the final couple of weeks. This appears to be due to a continued high level of data collection persistence and effort on the part of the Bandundu control group, which seems to be attributable to some intrinsic difference in this site compared to the other sites. In Kinshasa, incentivisation improved data collection efforts in the early parts of the trial compared to the control group, suggesting that at the time when participants were overall the most active, the incentive did improve the completeness of data collection efforts. However, this higher level of data collection effort by Kinshasa's incentive group dropped approximately halfway through the trial, after which there was not a significant difference between the control and incentive groups. This mirrors the results for participant activity, which showed that participation significantly dropped off halfway through. The decline in data collection efforts shows that even those who are continuing to participate are not collecting data for the entire time period (6 pm to 6 am) as requested in the data collection methodology. This may be in part due to the design of our incentives, which were given regardless of activity and were not scaled for completeness of participation. This result shows that despite incentivisation initially appearing to motivate complete data collection in one of our districts, the diminishing interest and effort over time are not overcome by incentivisation.

Our post-trial FGDs gave insight into issues surrounding incentives, including compensation and technology. Over half of the participants in the trial did not return for the post-trial FGDs. The reasons cited by the DRC team were a desire to keep the smartphone and the lack of payment at the control sites, though there were more post-trial FGD attendees from the control groups than the incentive groups. This compensation issue was emphasised by a refusal to return the phones by some participants in trial groups in both districts. The Bandundu control group exhibited higher phone retention

compared to the Bandundu incentive group, and the Kinshasa incentive group showed higher phone retention in contrast to the Kinshasa control group. There was no significant difference in the number of participants that named phone retention as a motive for participation between the districts, the trial groups, or between the trial groups within districts, showing that previously held values were not associated with phone retention. Instead, the pattern of phone retention mirrored the activity levels of each trial group, perhaps indicating that the more active participants felt a stronger entitlement to keeping the phones. However, phone retention was related to the relative effort in each district, rather than the overall effort. For example, the Bandundu incentive and the Kinshasa incentive had similar activity levels and total uploads, yet the Kinshasa incentive group retained twice the number of phones. As far as we are aware, participants in each district and trial group did not know the participation rates of the other groups, and therefore, did not know their relative participation. This indicates that individuals' participation might be influenced by their perception of effort.

The phone retention and refusal to return at the end of the study is an example of how citizen science projects can deviate from planned outcomes. In this context, participants effectively provided themselves with an incentive (or supplementary incentive) by refusing to return phones, despite study agreements and information about compensation ahead of the trial. A study on citizen science with specially designed video games found that game design can diminish citizen science outcomes and enhance the odds of cheating and cutting corners, depending on the ability to do so [59]. It is important to consider how incentive application design therefore may influence study outcomes and participation. The incentive group in this case received payment regardless of participation, which may have opened this study system to cutting corners (e.g., reduced activity and upload effort at the end of the study period). As an alternative, incentives given in response to data collection activity close this loophole and effectively encourage data collection persistence. Additionally, the post-trial survey in this study was not incentivised, and therefore participants gained more from refusing to return phones

than they did from attending. Balancing incentive value with requests and alternatives is necessary for incentives to operate as intended, and as seen in this study, some individuals will not follow prescriptive actions if they feel they can benefit in other ways.

Our post-trial FGDs revealed that despite there not being a significant or differential response to incentivisation, there were significant contrasting attitudes between the study groups in Bandundu and those in Kinshasa. Bandundu exhibited a significantly higher overall participation rate in FGDs compared to Kinshasa, and a greater proportion of Bandundu participants expressed willingness to partake in the trial again, irrespective of incentivisation (25 out of 26 post-trial FGD attendees). Out of 27 post-trial FGD attendees from Kinshasa, only 12 indicated a willingness to participate again. Additionally, there appear to be differences in values between the two districts. Among the 16 participants who identified knowledge as their motive for future participation, only one was from Kinshasa. Conversely, among Kinshasa participants who expressed willingness to participate again, the majority cited incentives as their driving factor. Further research is needed to understand how community and culture interact with incentive application, but in this case, despite differing motivations for participation, incentives did not have any significant effects on overall participation in the study.

The biggest issues cited by participants who took part in the trial were access to electricity to charge the smartphone sensor and internet connection. Interestingly, the groups that mentioned access to electricity significantly more were also the groups that performed best for data collection activity and effort in each district. It could be that the practicality of it was more apparent to the groups that put more effort in, and practical difficulties may be part of the reason people felt they should have been compensated better for the study. Of the incentive group participants who attended the focus group, over half said they felt they had not been given enough money for the study (14/22). This

dissatisfaction may have been due to the unexpected cost of transferring money, which reduced the originally stated compensation (from $10 to $9). In pre-trial FGDs, there were only six cases when participants who cited money as their motivation asked for a monetary value at or below the incentive value given in the study, suggesting that the incentive potentially fell below common expectations.

Despite the sentiment that the money was not enough, incentive group participants were more likely to say they would participate in the future than control group participants, and although bed nets were one of the least cited reasons for participating in the study, almost all participants mentioned the bed nets as a benefit they experienced in the trial, perhaps improving their desire to continue participation. Considering that all phones were turned on and collected data at least once during the study period, it appears useability was not a limiting factor of the study, and indeed all participants apart from one said that they felt the MozzWear app was easy to use. Due to the lack of practical issues with the study, our results highlight the importance of scaling expectations of citizen science and matching the value of incentives with the effort and length of trials to improve participation and satisfaction.

Our findings show that mobile applications using hardware on budget smartphones can generate useful data with limited interventions and suggests that mobile application development should be more widely considered in citizen science data collection efforts. Future research on the role of incentivization for data collection that utilizes technology should consider how to further disambiguate the inherent benefits in participation related to technological access, such as social status or monetary benefit from retaining equipment. We suggest that with increased mobile phone ownership, this complexity may be overcome by utilizing current phone owners, rather than providing participants with hardware. Additionally, as mobile phone use becomes more popular, opportunities for large scale studies will support more complex statistical approaches to account for the complex structures that

underpin intrinsic motivators and responses to extrinsic motivators, such as proximity to urban areas which are potentially obscured by the inferential statistical methods used in this study.

Conclusions

Results of this study show that in this study system incentives did not have significant effects on data collection activity throughout the trial but did have some effect on data collection persistence and effort, improving data collection effort during portions of the trial in one of our study districts. Our results indicate that community differences are what drive the trends in participation in this study system, and that more information is needed ahead of trials to assess how community differences may impact participation in citizen science data collection. Participant engagement with researchers and study organisers appears to improve data collection, particularly in longer studies. Communication with participants is important for appropriately scaling incentives to account for citizen data collection effort. Systems should be designed appropriately to maximise the alignment of incentives with active data collection. Overall, the incentives tested do not appear to significantly improve data collection in this study system, and in this case, increased engagement during data collection may have improved outcomes. With technology becoming an increasingly used tool in citizen science, its application should be considered in reference to existing infrastructure. Consideration should be made for the value that the technology represents in reference to the overall value of the incentive being offered to avoid issues with data sensor retention in response to changing attitudes towards incentive value.

Acknowledgements

Thank you to the DRC team at Kinshasa School of Public Health and University of Bandundu and to Paul Mansiangi, who has since passed away, for organising and co-ordinating the study. We are

grateful to the community leaders, health zone officials, and all the participants from Trois Rivières, Caravane, Bu and Mikondo for their support and participation in the study.

References

1. GSMA. GSMA | The Mobile Economy - Sub-Saharan Africa 2017 - GSMA Sub Saharan Africa [Internet]. 2017 [cited 2023 Aug 28]. Available from: https://www.gsma.com/subsaharanafrica/resources/mobile-economy-2017-sub-saharan-africa-2017
2. GSMA. GSMA | The State of Mobile Internet Connectivity Report 2019 | Mobile for Development [Internet]. 2019 [cited 2023 Aug 28]. Available from: https://www.gsma.com/mobilefordevelopment/resources/the-state-of-mobile-internet-connectivity-report-2019/
3. ITU Hub. Measuring digital development: Facts & figures 2019 - ITU Hub [Internet]. 2019 [cited 2023 Aug 28]. Available from: https://www.itu.int/hub/2020/05/measuring-digital-development-facts-figures-2019/
4. Baxter C, Carroll JA, Keogh B, Vandelanotte C. Assessment of Mobile Health Apps Using Built-In Smartphone Sensors for Diagnosis and Treatment: Systematic Survey of Apps Listed in International Curated Health App Libraries. JMIR Mhealth Uhealth. 2020 Feb 3;8(2):e16741.
5. Kamel Boulos MN, Brewer AC, Karimkhani C, Buller DB, Dellavalle RP. Mobile medical and health apps: state of the art, concerns, regulatory control and certification. Online J Public Health Inform. 2014 Feb 5;5(3).
6. Lee SH, Nurmatov UB, Nwaru BI, Mukherjee M, Grant L, Pagliari C. Effectiveness of mHealth interventions for maternal, newborn and child health in low– and middle–income countries: Systematic review and meta–analysis. J Glob Health [Internet]. 2016 [cited 2023 Aug 28];6(1). Available from: /pmc/articles/PMC4643860/

7. Lee Ventola C. Mobile Devices and Apps for Health Care Professionals: Uses and Benefits. Pharmacy and Therapeutics [Internet]. 2014 [cited 2023 Aug 28];39(5):356. Available from: /pmc/articles/PMC4029126/

8. Barrington J, Wereko-Brobby O, Ward P, Mwafongo W, Kungulwe S. SMS for Life: a pilot project to improve anti-malarial drug supply management in rural Tanzania using standard technology. Malar J. 2010 Dec 27;9(1):298.

9. Davis RG, Kamanga A, Castillo-Salgado C, Chime N, Mharakurwa S, Shiff C. Early detection of malaria foci for targeted interventions in endemic southern Zambia. Malar J [Internet]. 2011 Sep 12 [cited 2023 Aug 28];10(1):1–12. Available from: https://malariajournal.biomedcentral.com/articles/10.1186/1475-2875-10-260

10. Francis F, Ishengoma DS, Mmbando BP, Rutta ASM, Malecela MN, Mayala B, et al. Deployment and use of mobile phone technology for real-time reporting of fever cases and malaria treatment failure in areas of declining malaria transmission in Muheza district north-eastern Tanzania. Malar J [Internet]. 2017 Aug 1 [cited 2023 Aug 28];16(1). Available from: https://pubmed.ncbi.nlm.nih.gov/28764792/

11. Gibson DG, Ochieng B, Kagucia EW, Were J, Hayford K, Moulton LH, et al. Mobile phone-delivered reminders and incentives to improve childhood immunisation coverage and timeliness in Kenya (M-SIMU): a cluster randomised controlled trial. Lancet Glob Health [Internet]. 2017 Apr 1 [cited 2023 Aug 28];5(4):e428–38. Available from: https://pubmed.ncbi.nlm.nih.gov/28288747/

12. Kamanga A, Moono P, Stresman G, Mharakurwa S, Shiff C. Rural health centres, communities and malaria case detection in Zambia using mobile telephones: A means to detect potential reservoirs of infection in unstable transmission conditions. Malar J [Internet]. 2010 Apr 15 [cited 2023 Aug 28];9(1):1–7. Available from: https://malariajournal.biomedcentral.com/articles/10.1186/1475-2875-9-96

13. Kaunda-Khangamwa BN, Steinhardt LC, Rowe AK, Gumbo A, Moyo D, Nsona H, et al. The effect of mobile phone text message reminders on health workers' adherence to case management guidelines for malaria and other diseases in Malawi: lessons from qualitative data from a cluster-randomized trial. Malar J [Internet]. 2018 Dec 19 [cited 2023 Aug 28];17(1). Available from: https://pubmed.ncbi.nlm.nih.gov/30567603/

14. Meankaew P, Kaewkungwal J, Khamsiriwatchara A, Khunthong P, Singhasivanon P, Satimai W. Application of mobile-technology for disease and treatment monitoring of malaria in the "Better Border Healthcare Programme." Malar J [Internet]. 2010 Aug 19 [cited 2023 Aug 28];9(1):1–14. Available from: https://link.springer.com/articles/10.1186/1475-2875-9-237

15. Wakadha H, Chandir S, Were EV, Rubin A, Obor D, Levine OS, et al. The feasibility of using mobile-phone based SMS reminders and conditional cash transfers to improve timely immunization in rural Kenya. Vaccine [Internet]. 2013 Jan 30 [cited 2023 Aug 28];31(6):987–93. Available from: https://pubmed.ncbi.nlm.nih.gov/23246258/

16. Roche J, Bell L, Galvão C, Golumbic YN, Kloetzer L, Knoben N, et al. Citizen Science, Education, and Learning: Challenges and Opportunities. Frontiers in Sociology. 2020 Dec 2;5:613814.

17. Conrad CC, Hilchey KG. A review of citizen science and community-based environmental monitoring: Issues and opportunities. Environ Monit Assess [Internet]. 2011 May 17 [cited 2023 Oct 2];176(1–4):273–91. Available from: https://link.springer.com/article/10.1007/s10661-010-1582-5

18. Den Broeder L, Devilee J, Van Oers H, Schuit AJ, Wagemakers A. Citizen Science for public health. Health Promot Int [Internet]. 2018 Jun 1 [cited 2023 Oct 2];33(3):505–14. Available from: https://dx.doi.org/10.1093/heapro/daw086

19. Di Domenico SI, Ryan RM. The emerging neuroscience of intrinsic motivation: A new frontier in self-determination research. Front Hum Neurosci. 2017 Mar 24;11:247005.

20. Hennessey B, Moran S, Altringer B, Amabile TM. Extrinsic and Intrinsic Motivation. Wiley Encyclopedia of Management. 2015 Jan 21;1–4.

21. Ryan RM, Deci EL. Intrinsic and extrinsic motivation from a self-determination theory perspective: Definitions, theory, practices, and future directions. Contemp Educ Psychol. 2020 Apr 1;61.

22. Aceves-Bueno E, Adeleye AS, Bradley D, Tyler Brandt W, Callery P, Feraud M, et al. Citizen Science as an Approach for Overcoming Insufficient Monitoring and Inadequate Stakeholder Buy-in in Adaptive Management: Criteria and Evidence. Ecosystems [Internet]. 2015 Apr 1 [cited 2023 Oct 2];18(3):493–506. Available from: https://link.springer.com/article/10.1007/s10021-015-9842-4

23. Cetas ER, Yasué M. A systematic review of motivational values and conservation success in and around protected areas. Conserv Biol [Internet]. 2017 Feb 1 [cited 2023 Aug 28];31(1):203–12. Available from: https://pubmed.ncbi.nlm.nih.gov/27254293/

24. Lester RT, Ritvo P, Mills EJ, Kariri A, Karanja S, Chung MH, et al. Effects of a mobile phone short message service on antiretroviral treatment adherence in Kenya (WelTel Kenya1): a randomised trial. Lancet [Internet]. 2010 Nov 27 [cited 2023 Aug 28];376(9755):1838–45. Available from: https://pubmed.ncbi.nlm.nih.gov/21071074/

25. Ngabo F, Nguimfack J, Nwaigwe F, Mugeni C, Muhoza D, Wilson DR, et al. Designing and Implementing an Innovative SMS-based alert system (RapidSMS-MCH) to monitor pregnancy and reduce maternal and child deaths in Rwanda. Pan Afr Med J [Internet]. 2012 [cited 2023 Aug 28];13. Available from: /pmc/articles/PMC3542808/

26. Richter A, Comay O, Svenningsen CS, Larsen JC, Hecker S, Tøttrup AP, et al. Motivation and support services in citizen science insect monitoring: A cross-country study. Biol Conserv. 2021 Nov 1;263.

27. Tomlinson M, Solomon W, Singh Y, Doherty T, Chopra M, Ijumba P, et al. The use of mobile phones as a data collection tool: a report from a household survey in South Africa. BMC Med Inform Decis Mak [Internet]. 2009 [cited 2023 Aug 28];9(1). Available from: https://pubmed.ncbi.nlm.nih.gov/20030813/

28. Bell S, Marzano M, Cent J, Kobierska H, Podjed D, Vandzinskaite D, et al. What counts? Volunteers and their organisations in the recording and monitoring of biodiversity. Biodivers Conserv. 2008 Dec 26;17(14):3443–54.

29. Finkelstien MA. Intrinsic vs Extrinsic Motivational Orientations and the Volunteer Process. Pers Individ Dif [Internet]. 2009 Jan 1 [cited 2023 Aug 28];46(5–6):653. Available from: https://digitalcommons.usf.edu/psy_facpub/770

30. Hobbs SJ, White PCL. Motivations and barriers in relation to community participation in biodiversity recording. J Nat Conserv [Internet]. 2012 Dec [cited 2023 Aug 28];20(6):364–73. Available from: https://pure.york.ac.uk/portal/en/publications/motivations-and-barriers-in-relation-to-community-participation-i

31. Jordan Raddick M, Bracey G, Gay PL, Lintott CJ, Cardamone C, Murray P, et al. Galaxy zoo: Motivations of citizen scientists. Astronomy Education Review. 2013;12(1).

32. Cerasoli CP, Nicklin JM, Ford MT. Intrinsic motivation and extrinsic incentives jointly predict performance: A 40-year meta-analysis. Psychol Bull. 2014;140(4):980–1008.

33. Patall EA, Cooper H, Robinson JC. The effects of choice on intrinsic motivation and related outcomes: a meta-analysis of research findings. Psychol Bull [Internet]. 2008 Mar [cited 2023 Aug 28];134(2):270–300. Available from: https://pubmed.ncbi.nlm.nih.gov/18298272/

34. Dam R, Mponzi W, Msaky D, Mwandyala T, Kaindoa EW, Sinka ME, et al. What incentives encourage local communities to collect and upload mosquito sound data by using smartphones? A mixed methods study in Tanzania. Glob Health Res Policy. 2023 Dec 1;8(1).

35. Sinka ME, Zilli D, Li Y, Kiskin I, Msaky D, Kihonda J, et al. HumBug – An Acoustic Mosquito Monitoring Tool for use on budget smartphones. Methods Ecol Evol [Internet]. 2021 Oct 1 [cited 2023 Aug 28];12(10):1848–59. Available from: https://onlinelibrary.wiley.com/doi/full/10.1111/2041-210X.13663

36. Kiskin I, Zilli D, Li Y, Sinka M, Willis K, Roberts S. Bioacoustic detection with wavelet-conditioned convolutional neural networks. Neural Comput Appl. 2020 Feb 1;32(4):915–27.

37. Li Y, Kiskin I, Zilli D, Sinka M, Chan H, Willis K, et al. Cost-sensitive detection with variational autoencoders for environmental acoustic sensing. 2017 Dec 6 [cited 2023 Dec 7]; Available from: http://arxiv.org/abs/1712.02488

38. Kiskin I, Orozco BP, Windebank T, Zilli D, Sinka M, Willis K, et al. Mosquito Detection with Neural Networks: The Buzz of Deep Learning. 2017 May 15 [cited 2023 Dec 7]; Available from: http://arxiv.org/abs/1705.05180

39. Li Y, Zilli D, Chan H, Kiskin I, Sinka M, Roberts S, et al. Mosquito detection with low-cost smartphones: data acquisition for malaria research. 2017 Nov 16 [cited 2023 Dec 7]; Available from: http://arxiv.org/abs/1711.06346

40. Kiskin I, Cobb AD, Sinka M, Willis K, Roberts SJ. Automatic Acoustic Mosquito Tagging with Bayesian Neural Networks. Lecture Notes in Computer Science (including subseries Lecture Notes in Artificial Intelligence and Lecture Notes in Bioinformatics). 2021;12978 LNAI:351–66.

41. Kiskin I, Cobb AD, Wang L, Roberts S. Humbug Zooniverse: A Crowd-Sourced Acoustic Mosquito Dataset. ICASSP, IEEE International Conference on Acoustics, Speech and Signal Processing - Proceedings. 2020 May 1;2020-May:916–20.

42. Li Y, Kiskin I, Sinka M, Zilli D, Chan H, Herreros-Moya E, et al. Fast mosquito acoustic detection with field cup recordings: an initial investigation. dcase.community [Internet]. 2018 [cited 2024 Aug 16]; Available from: https://dcase.community/documents/workshop2018/proceedings/DCASE2018Workshop_Li_136.pdf

43. Centers for Disease Control and Prevention. CDC in the Democratic Republic of the Congo | Global Health | CDC [Internet]. 2023 [cited 2023 Aug 28]. Available from: https://www.cdc.gov/globalhealth/countries/drc/default.htm

44. World Health Organisation. World malaria report 2022 [Internet]. 2022 [cited 2023 Aug 28]. Available from: https://www.who.int/teams/global-malaria-programme/reports/world-malaria-report-2022

45. SimpleMaps.com. "Democratic Republic of the Congo (DRC) GIS Data." *SimpleMaps.com*, 2024, https://simplemaps.com/gis/country/cd#admin1. Accessed 16 Oct. 2024. Licensed under Creative Commons Attribution License. https://creativecommons.org/licenses/by/4.0/

46. Center for International Earth Science Information Network (CIESIN), Columbia University, Ministère de la Santé Publique, Hygiène et Prévention, Democratic Republic of the Congo, and GRID3. (2025). *GRID3 COD - Health Areas v4.0: Kinshasa health areas (GeoPackage)*. New York: Columbia University. https://data.humdata.org/dataset/grid3-cod-health-areas-v4-0. Accessed April 3, 2025. Licensed under Creative Commons Attribution License. https://creativecommons.org/licenses/by/4.0/

47. Center for International Earth Science Information Network (CIESIN), Columbia University, Ministère de la Santé Publique, Hygiène et Prévention, Democratic Republic of the Congo, and GRID3. (2025). *GRID3 COD - Health Areas v4.0: Kwilu health areas (GeoPackage)*. New York: Columbia University. https://data.humdata.org/dataset/grid3-cod-health-areas-v4-0. Accessed April 3, 2025. Licensed under Creative Commons Attribution License. https://creativecommons.org/licenses/by/4.0/

48. QGIS Development Team. QGIS [3.0.0]. Open Source Geospatial Foundation; 2018. Available from: https://qgis.org/en/site/.

49. Saldaña Johnny. The Coding Manual for Qualitative Researchers. 2021;1–440.

50. R Core Team. R: A Language and Environment for Statistical Computing_. 2023; R Foundation for Statistical Computing, Vienna, Austria. <https://www.R-project.org/>.

51. Kruskal WH, Wallis WA. Use of ranks in one-criterion variance analysis. J Am Stat Assoc. 1952;47:583-621. Available from: http://dx.doi.org/10.1080/01621459.1952.10483441.

52. Dinno A. dunn.test: Dunn's Test of Multiple Comparisons Using Rank Sums. R package. 2024. Available from: <https://CRAN.R-project.org/package=dunn.test>.

53. Wickham H. ggpolt2 Elegant Graphics for Data Analysis. Use R! series. 2016;211.

54. Kerr JM, Bum T, Lapinski MK, Liu RW, Lu Z, Zhao J. The effects of social norms on motivation crowding: experimental evidence from the Tibetan Plateau. Int J Commons [Internet]. 2019 [cited 2023 Aug 28];13(1):430–54. Available from: https://thecommonsjournal.org/articles/10.18352/ijc.882

55. Rode J, Gómez-Baggethun E, Krause T. Motivation crowding by economic incentives in conservation policy: A review of the empirical evidence. Ecological Economics. 2015 Sep 1;117:270–82.

56. Aceves-Bueno E, Adeleye AS, Feraud M, Huang Y, Tao M, Yang Y, et al. The Accuracy of Citizen Science Data: A Quantitative Review. Bulletin of the Ecological Society of America. 2017 Oct;98(4):278–90.

57. Peter M, Diekötter T, Kremer K. Participant Outcomes of Biodiversity Citizen Science Projects: A Systematic Literature Review. Sustainability 2019, Vol 11, Page 2780 [Internet]. 2019 May 15 [cited 2023 Oct 1];11(10):2780. Available from: https://www.mdpi.com/2071-1050/11/10/2780/htm

58. Ganzevoort W, van den Born RJG, Halffman W, Turnhout S. Sharing biodiversity data: citizen scientists' concerns and motivations. Biodivers Conserv [Internet]. 2017 Nov 1 [cited 2023 Oct 1];26(12):2821–37. Available from: https://link.springer.com/article/10.1007/s10531-017-1391-z

59. Prestopnik N, Crowston K. Purposeful gaming & socio-computational systems. In: Proceedings of the 17th ACM international conference on Supporting group work. New York, NY, USA: ACM; 2012. p. 75–84.

Supplementary Information

S1 Appendix: The DHS Demographic Survey Administered to Participants

S2 Appendix: The focus group discussion questions administered pre-trial and post-trial.

S3 Table: A table of results from comparisons of the demographic survey between study districts, trial groups overall, and trial groups within each district.

Figure 5

Click here to access/download;Figure;Fig5.tif

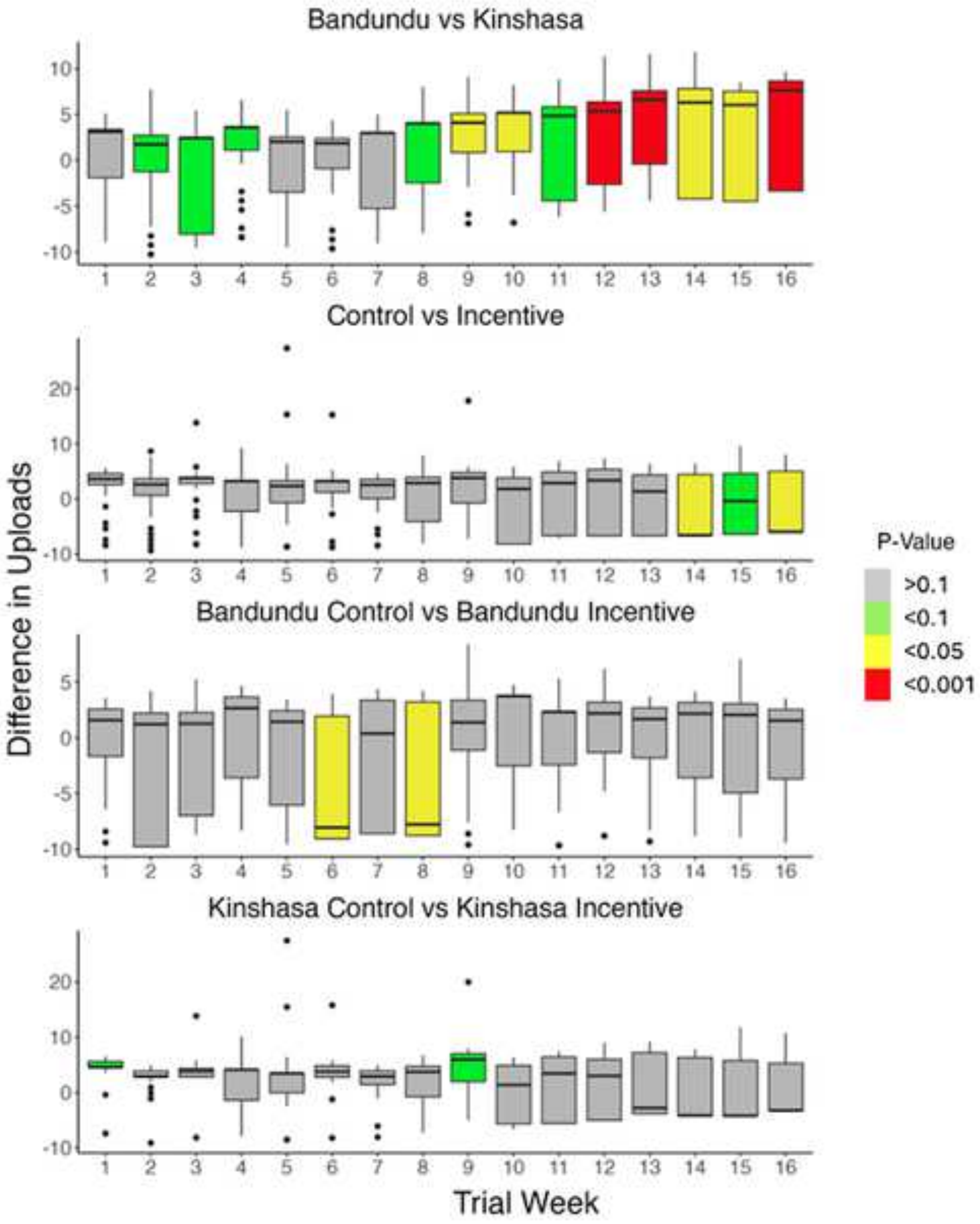

Figure 4

Click here to access/download;Figure;Fig4.tif

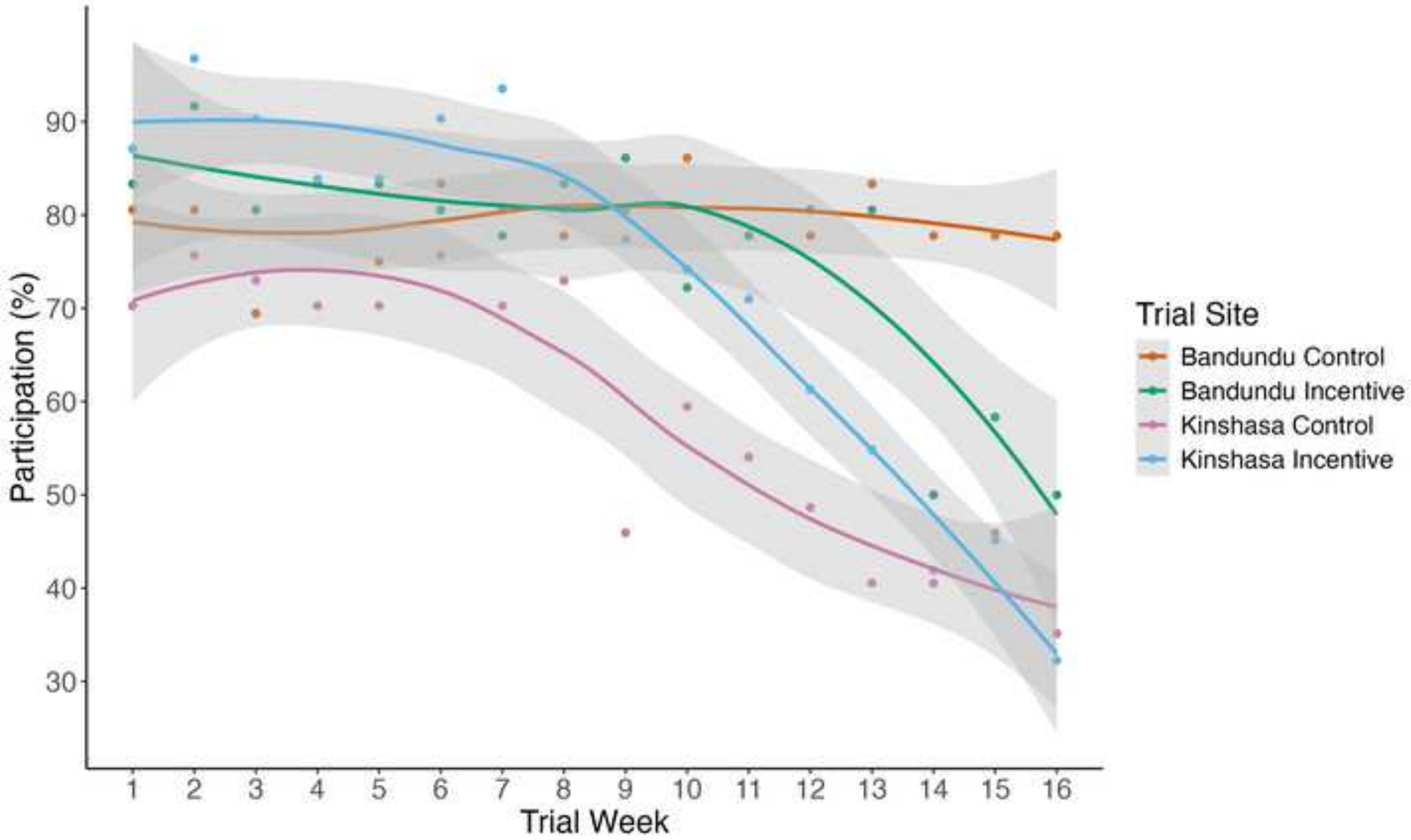

Figure 3

Click here to access/download;Figure;Fig3.tif

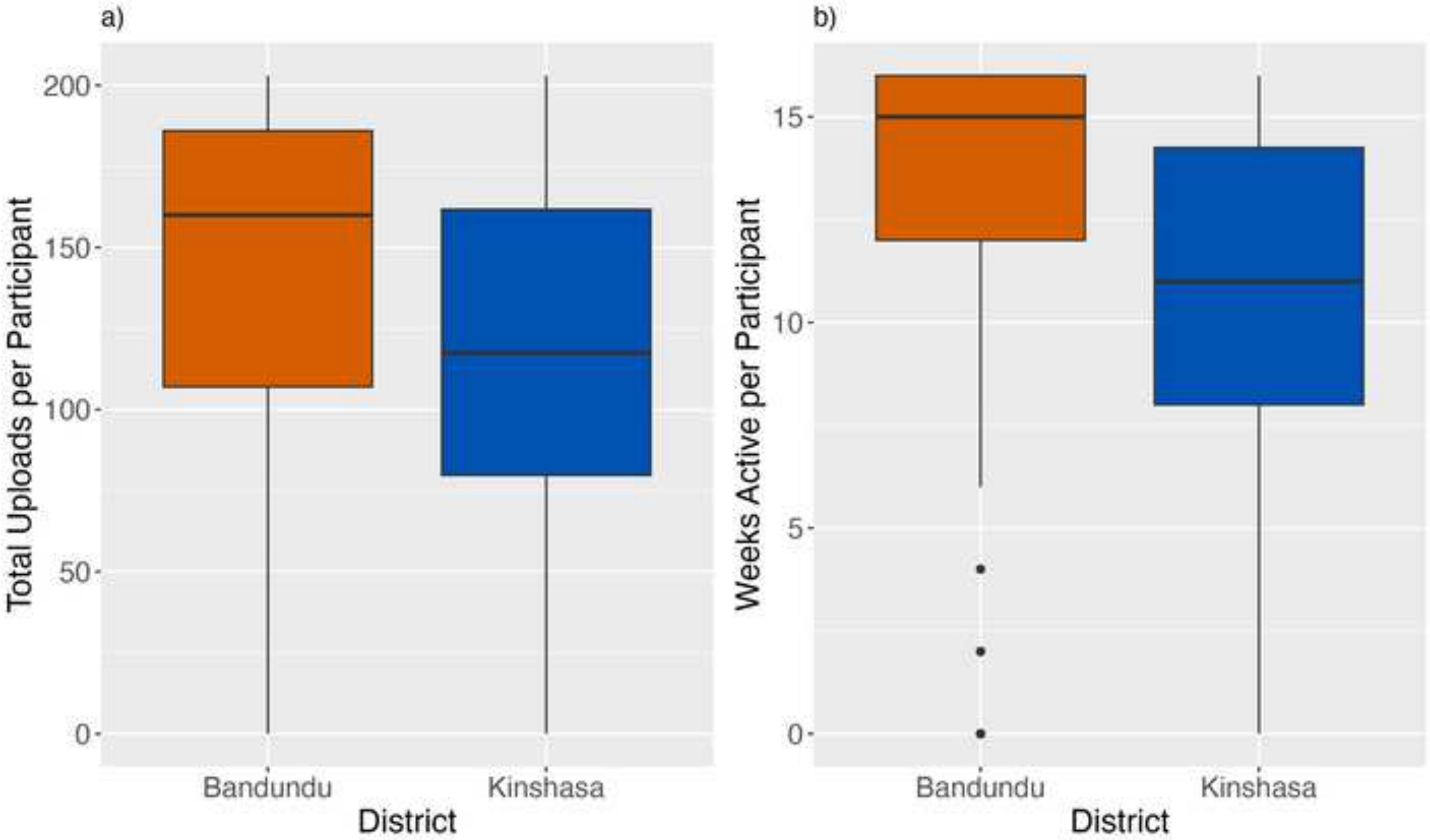

Figure 2

Click here to access/download;Figure;Fig2.tif

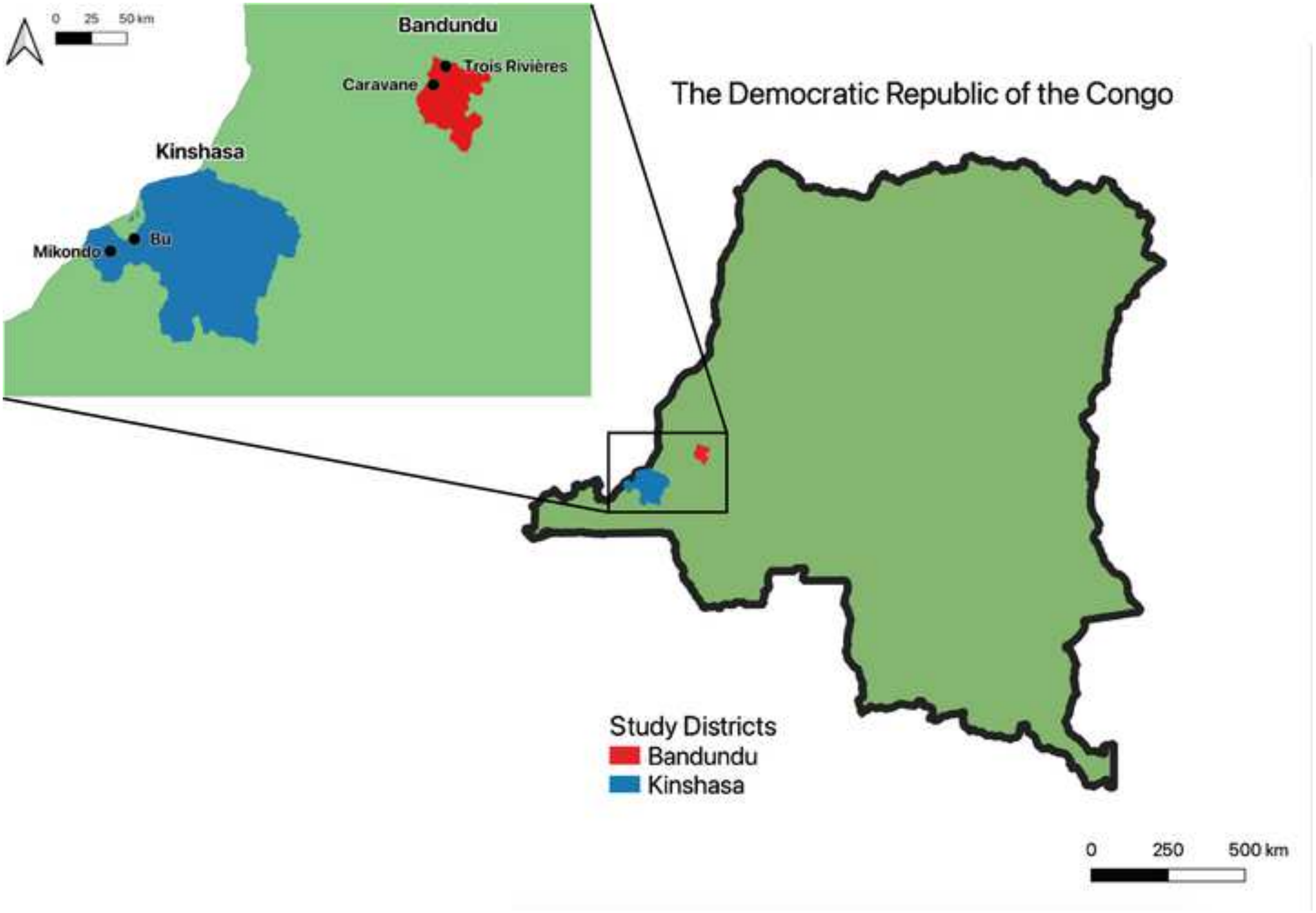

Figure 1

Click here to access/download;Figure;Fig1.tif

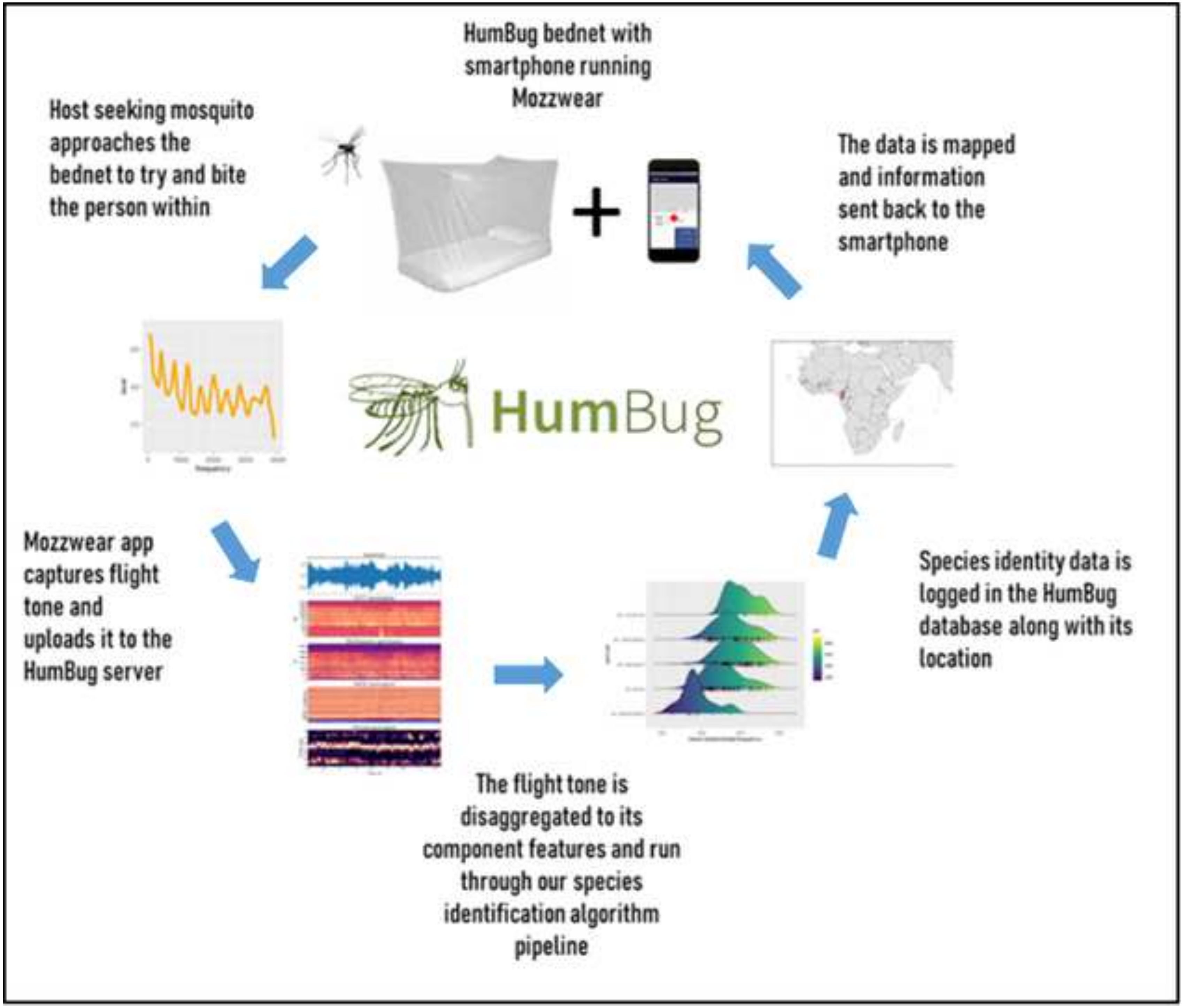

Click here to access/download
Supporting Information
Storer.et.al.DRC.Supp.docx

~~What~~ Do monetary incentives encourage local communities to collect and upload mosquito sound data ~~by~~ using smartphones? A case study in the Democratic Republic of the Congo

Short Title: Incentives for citizen science data collection in the DRC

Kieran E. Storer[1*], Jane P. Messina[2], Eva Herreros-Moya[1], Emery Metelo[3,4]¶, Josué Zanga[4]¶, Nono M. Mvuama[4]¶, Soleil Muzinga[4]¶, Rinita Dam[5]&, Marianne Sinka[1]&, Ivan Kiskin[6]&, Josh Everett[1]&, Yunpeng Li[7]&, Stephen Roberts[8]&, and Katherine J. Willis[1]

Affiliations

[1] Department of Biology, University of Oxford, Oxford, United Kingdom

[2] School of Geography and the Environment, Oxford University Centre for the Environment, University of Oxford, Oxford, United Kingdom

[3] Department of Entomology, National Institute for Biomedical Research, ~~M8R2+4WX,~~ Kinshasa, Democratic Republic of the Congo

[4] Kinshasa School of Public Health, University of Kinshasa, ~~H8Q3+2HV,~~ Kinshasa, Democratic Republic of the Congo

[5] Warwick Medical School, University of Warwick, Coventry, United Kingdom

[6] Surrey Institute for People-Centred AI, Centre for Vision Speech and Signal Processing, University of Surrey, Guildford, United Kingdom

[7] Centre for Oral, Clinical, and Translational Sciences, King's College London, London, United Kingdom

[8] Department of Engineering Science, University of Oxford, Oxford, United Kingdom

Formatted: Font: (Default) Times New Roman

Formatted: Font: (Default) Times New Roman

Formatted: Font: (Default) Times New Roman

Formatted: Font: (Default) Times New Roman

Formatted: Font: (Default) Times New Roman

Formatted: Superscript

Formatted: Font: (Default) Times New Roman

Formatted: Font: (Default) Times New Roman

Formatted: Font: (Default) Times New Roman

Formatted: Font: (Default) Times New Roman

Formatted: Font: (Default) Times New Roman

Formatted: Font: (Default) Times New Roman

Formatted: Font: (Default) Times New Roman

*Corresponding Author

*Corresponding Author

E-mail: kieran.storer@biology.ox.ac.uk (KES)

Phone: +447851099304

[¶] EM, JZ, NMM, and SM contributed equally to this work

[&] RD, MS, IK, JE, YL, and SR also contributed equally to this work

Formatted: Font: (Default) Times New Roman, No underline

Formatted: Hyperlink, Font: (Default) Times New Roman, 11 pt

Formatted: Font: (Default) Times New Roman

Field Code Changed

Formatted: No underline, Font color: Auto, Superscript

Formatted: Superscript

Attribution

KES contributed to data curation, formal analysis, methodology, visualization, and writing the original draft. JPM contributed to formal analysis and visualization. EHM contributed to conceptualization, investigation, methodology, project administration, supervision, visualization, and the review and editing of the manuscript. EM, JZ, NMM, and SM contributed to conceptualization, investigation, methodology, data collection, and review and editing of the manuscript. RD, MS, IK, JE, YL, and SR contributed to conceptualization, methodology, and review and editing of the manuscript. KJW contributed to funding acquisition, methodology, supervision, and the review and editing of the manuscript.

Formatted: Font: (Default) Times New Roman, No underline, Font color: Auto

Formatted: Font: (Default) Times New Roman

Formatted: Font: (Default) Times New Roman

Funding

This study was funded by the Bill and Melinda Gates Foundation Grant Award Number: OPP1209888.

Abstract

Formatted: Heading 1, Line spacing: single

Malaria is one of the deadliest vector borne diseases aeffecting sub-Saharan Africa. A suite of systems are being used to monitor and manage malaria risk and disease incidence, with an increasing focus on technological interventions that allow private citizens to remotely record and upload data. However, data collected by citizen scientists must be standardised and consistent if it is to be used for scientific analysis. Studies that aim to improve data collection quality and quantity have often included incentivisation, providing citizen scientists with monetary or other benefits for their participation in data collection. We tested whether monetary incentives enhance participation and data collection in a study trialling an acoustic mosquito sensor. Working with the community in two health districts in the Democratic Republic of Congo, we measured data collection participation, completeness, and community responses. Our results showed mixed responses to the incentive, with more participants interested in the social status and monetary value of the technology used than the monetary incentive itself. The effect of incentives on data collection varied over the course of the trial, increasing participation in the start of the trial but with no effect in the latter half of the trial. Feedback from participants showed that opinions on technology, research objectives, and incentives varied between communities, and was associated with differences in data collection quantity and quality, suggesting that differences in community interest in data collection and the incentives may be more important than the incentive value itself. These results suggest that though there is an initial benefit, extrinsic motivations do not override differences in intrinsic motivations over time, and enhanced communication and dialogue with participants may improve citizen science participation and attitudes.

Introduction

Formatted: Heading 1, Line spacing: single

Mobile phone usage in sub-Saharan Africa reached 489 million users in 2023, with smartphones making up 51% of mobile phones due to improvements in mobile phone access and network connections ([1–3]). With increasing smartphone ownership, their use for citizen science data

collection has also increased in tandem, making use of the built-in camera, sensors, and GPS to generate and record data ~~(~~[4–7]~~)~~. Citizen science projects have ranged widely, and have been increasingly used in healthcare settings, including for monitoring diseases such as malaria by tracking outbreaks, monitoring drug stocks, reminding patients of medical appointments, and recording mosquito larval habitats and populations ~~(~~[for examples see: 8–15]~~)~~. As the amount and type of data collected by private citizens increases there are opportunities for developing high-resolution data repositories that widen citizen participation to include a greater range of demographic groups.

Previous research on citizen science data collection has shown that gathering data for research or healthcare purposes can help to improve educational outcomes ~~(~~[16]~~)~~, make data collectors feel more connected to their community ~~(~~[17],~~),~~ and improve health outcomes ~~(~~[18]~~)~~. But how do researchers motivate people to participate and collect high-quality data? Firstly, there is need to understand the roles that intrinsic and extrinsic motivations play in citizen scientist participation and data collection quality. Intrinsic motivation factors are 'people's spontaneous tendencies to be curious and interested, to seek out challenges and to exercise and develop their skills and knowledge, even in the absence of operationally separable rewards' ~~(~~[19]~~)~~. Intrinsic motivation is influenced by local and regional cultures, education, and familial values ~~(~~[20]~~)~~. In contrast, extrinsic motivators are 'behaviours done for reasons other than their inherent satisfactions' ~~(~~[21]~~)~~, such as monetary gain ~~(~~[22]~~)~~. Efforts to improve data collection typically target these different motivation factors. Studies have shown that monetary incentives can be used in health care and ecological monitoring schemes to improve participation ~~(~~[23–27]~~)~~ and that intrinsic factors can be harnessed to improve data collection participation and quality by improving learning opportunities and using participant knowledge ~~(~~[28–31]~~)~~.

The complex spatiotemporal variability inherent to motivation factors ([32,33]) and the theoretical potential for a trade-off between them ([21]) make understanding their influence essential to improving data collection quality and scope while optimising resource allocation. To study participants' motivations and assess the functionality of incentive applications to promote community involvement in the collection of high-quality long-term biological data, we previously ran a data collection trial using incentives and SMS text reminders for collecting mosquito audio data in Tanzania using the HumBug tool ([34]). This tool is a smartphone audio sensor combined with a modified bed net designed to temporarily trap mosquitoes and guide them towards a budget smartphone running the MozzWear app that records the flight tone of host seeking mosquitoes overnight to determine the abundance and diversity of mosquitoes present using their sound ([35]) ((Fig. 1).; For details on the Humbug tool, also see ([36–42]).

Fig. 1: HumBug Tool configuration, showing the data collection, upload, and analysis pipeline (reproduced from Sinka et al., 2021).

The smartphone app MozzWear used in the HumBug tool records mosquito flight tone data overnight and provides a secure connection to a server at the University of Oxford where the data is uploaded via the mobile or Wi-Fi data network. The data then runs through an algorithm pipeline that first detects the mosquito flight tone from background noise and then identifies the mosquito species using their acoustic signature ([35–37,40]). As such, the HumBug tool is a good example of using smartphone technologies to provide an accessible biological data collection methodology to identify and monitor vectors of human disease - in this case, mosquitoes.

Previous work in Tanzania showed that providing homeowners with monetary incentives and SMS reminders to record and upload mosquito data did not significantly increase the number of uploads when compared to the control group ([34]). Instead, the study found that other factors influenced data

collection efforts. However, it did not examine in detail what these other factors might be. To better understand, therefore, how a monetary extrinsic factor~~s~~ may affect data collection practices in the use of the HumBug tool, we ran a second data collection trial in a different community within two districts of the Democratic Republic of the Congo: Bandundu, and rural Kinshasa. In this study we aimed to test whether monetary extrinsic incentives encouraged: i) participant data collection activity during the trial period; ii) participant effort (the number of uploads per participant made during the sampling period, indicative of following trial protocols); and iii) the persistence of participation over time (whether trial participants continued to upload data throughout the sampling period). To address these questions, we compared participant activity (weeks active) and sampling effort (number of uploads) over the sampling period to assess differences in participation associated with receiving incentives for data collection. This study builds upon our previous work in Tanzania to fill gaps in understanding how monetary incentives alone influence the consistency and quality of audio data collection.

Methods

Formatted: Heading 1, Line spacing: single

The study ran from April to November of 2022 in two districts of the Democratic Republic of the Congo (DRC): rural Kinshasa, and Bandundu. Our collaborators at the University of Kinshasa and the University of Bandundu selected participants, conducted demographic surveys and pre- and post-trial focus group discussions, and ran the study. Data analysis and writing of the manuscript were carried out by the University of Oxford team. All leaders of communities, health zone officials, and participants provided signed consent forms for the study, and the study was approved by the University of Oxford Tropical Research Ethics Committee and the University of Kinshasa Public Health Ethics Committee. Informed written consent was obtained from all interviewees prior to the focus group discussions and the quantitative feedback survey. Consent forms signed by all the participants included the release of summary findings and details of individual responses from this study. Potential participants were informed of the voluntary nature of the study and had at least 24 hours to consider

Formatted: Justified, Line spacing: Double

taking part. Efforts were made to create a safe place for sharing experiences during the focus group discussions.

Study Locations

Geographically, the DRC covers an area of approximately 2,345,409 km². The country is dominated by the Congo River basin surrounded by high plateaus, resulting in high precipitation and thick tropical forest within the basin and grassland in the plateaus above. In the DRC, malaria is a major cause of illness and death. Worldwide, 12% of the total malaria cases occurred in the DRC in 2022, causing 60% of hospital visits in the country ~~(~~[43,44]~~)~~.

Kinshasa is the capital city of the DRC and the largest city in central Africa. With ~16 million population it is the third largest mega-city on the continent. The Bandundu district has a total of 143,435 people and covers an area of 222 Km² (Figure 2). Participants in our research were from the Bu and Mikondo health districts of Kinshasa, and the Trois Rivières and Caravane health districts of Bandundu.

Fig. 2: Map of the Democratic Republic of Congo, modified from simplemaps.com [45] and GRID3 COD - Health Areas v4.0 [46,47] *under CC BY 4.0 license, -showing the ~~provinces of the DRC (45), the location of the trial districts (46)~~ location of study districts; ~~showing~~ Bandundu in red~~purple~~ and Kinshasa in blue~~orange~~. On the zoom inset, ~~, with~~ black~~red~~ points show~~wing~~ the location of study sites ~~in the zoom inset~~. Mapping was carried out ~~, all visualised~~ using QGIS 3.30.0 ~~(~~[48~~7~~]~~)~~.*

Each district in our study had two trial sites: a control site and an incentive site. The selection of participants from our four trial sites took place from April 2 to June 30, 2022. In each health district, authorities of the different Health Zones, the village chiefs, and heads of districts were contacted to

Formatted: Font: (Default) Times New Roman, English (United States)

Formatted: Font: (Default) Times New Roman

Formatted: Heading 2, Line spacing: single

Formatted: Default Paragraph Font, Font: (Default) Times New Roman, Font color: Gray-65%

Formatted: Font: (Default) Times New Roman

Formatted: Default Paragraph Font, Font: (Default) Times New Roman, Font color: Gray-65%

Formatted: Font: (Default) Times New Roman

Formatted: Default Paragraph Font, Font: (Default) Times New Roman, Font color: Gray-65%

Formatted: Font: (Default) Times New Roman

Formatted: Font: (Default) Times New Roman, 11 pt, Italic, Font color: Gray-65%

Formatted: Font: (Default) Times New Roman

gain permission for the study and to involve local leadership. Meetings with local leaders were carried out to plan the recruitment of households for the study, and to allocate which health districts would be the control treatment (Trois Rivières in Bandundu and Bu in Kinshasa) and the incentive treatment (Caravane in Bandundu and Mikondo in Kinshasa). These health districts will hereafter be referred to as a trial sites for clarity.

Households were recruited using a modified random walk technique from an entry point chosen from the main entrances to the village/street selected by local health district officials. To reduce the bias of entry point selection, each household was approached sequentially along the street ~~From the entry point, the selection group~~and ~~-~~asked if they wanted to participate, ~~progressed~~ until the number of households required to participate at the site (n=37) was met. Trial participants were admissible if they were 18+ years old, owned or had access to a personal mobile phone, were residents in Bandundu/Kinshasa throughout the study, and were willing and capable of providing a signed consent form.

Ahead of the trial, training of the interviewers and moderators took place over a day in each of the trial sites to teach local health officials and local leadership how to run interviews and support participants in the trial. Demonstrators were also taught how to install and use the HumBug tool and MozzWear App and were given a presentation of the trial objectives. ~~(Figures 3 and 4).~~ Finally, interviewers were trained on how to administer the demographic questionnaires to participants who agreed to take part in the trial (DHS Phase 8 Questionnaire, S1 Appendix).

~~*Fig. 3: Images of the DRC showing trial participants how to record and upload data using the Humbug tool.*~~

~~*Fig. 4: Meetings with the participants to show them how to set up the HumBug net.*~~

Moderators ran pre-trial focus group discussions (FGDs) to confirm that participants understood the purpose of the study, how the study was designed, and how to operate the HumBug tool and MozzWear application. The pre-trial FGDs were also used to discuss and understand the challenges that participants may face, their motivations for taking part in the study, and what they thought the impact would be on their lives. The pre- and post- trial FGD questions can be found in the S2 Appendix. The reasons participants gave for joining the study were categorized into common themes of access to electricity, financial incentive, use of a phone, provision of bed nets, contributing to health improvements and malaria control, or gaining personal knowledge. Participant FGDs responses were then structurally coded for analysis ([498]).

Control group participants were provided with the HumBug tool (a smartphone running the MozzWear app and the HumBug bed net), and one dollar to pay for an internet connection. The incentive group was provided with the HumBug tool and one dollar for an internet connection, and an additional ten dollars each month paid via airtime to their mobile phones. Participants were instructed to place the smartphone with the MozzWear app in the HumBug net and start the record function at 18:00 hrs and turn it off at 06:00 hrs on their allocated weekly recording day during the trial period (16 weeks total). Recordings were split at one-hour intervals automatically in the MozzWear app to prepare recordings for algorithmic analysis. As such a complete recording effort would show 12 recordings. The recordings were then uploaded by participants to the remote server when they connected to the internet. Once participants were taught how to use the Humbug tool there was no contact between the research team and the participants.

The research team conducted post-trial FGDs to understand the participants' experience of using the HumBug tool and participating in the study. Questions included whether they liked using the MozzWear app, whether they liked using the HumBug net, how the trial personally affected them,

what they would want to be different about the trial in the future, whether they would participate in the future, and if they received an incentive whether it was enough money. Data from the post-trial FGDs was coded for analysis by themes of response to questions as described for the pre-trial FGDs.

Data Analysis

To compare the effect of providing incentives to the participants to upload mosquito audio data, the number of active participants (participants who uploaded any data) and the number of uploads were counted during each trial week. Participant identification numbers were used to assess the upload counts and weekly activity by location (Kinshasa or Bandundu districts) and experimental group (control or incentive). Counts of uploads, weeks of participation, and the average number of participants each week were compared using Wilcoxon Rank Sum tests ~~(~~[50~~49~~]~~)~~ and Kruskal-Wallis tests ~~(~~[51~~0~~]~~)~~ with a post-hoc Dunn test ~~(~~[52~~1~~]~~)~~ to assess if there was a significant difference in participation between the experimental groups/locations over the study period and reduce potential type 1 error, associated with multiple comparisons. The number of participant uploads was broken down for each week of the trial to assess whether incentives improved data collection persistence throughout the study and was also compared using Wilcoxon Rank Sum Tests. Data on income, sex, age, education, and profession were also evaluated as explanatory variables to explain variation in data collection between groups. Comparisons of the demographic data of each district, trial group, and combination of the two, were made using Wilcoxon Rank Sum tests and Chi-squared tests. The pretrial FGD data was analysed using Wilcoxon Rank Sum tests to compare differences in pre-trial motivations for participation. The post-trial FGD data was analysed using a Fisher's Exact Test to assess the correlation between district and trial groups on attendance for the post-trial FGD and whether participants would take part in a future trial, and phone return. All plots were produced using ggplot2 ~~(~~[53~~2~~]~~)~~.

Formatted: Heading 2, Line spacing: single

Formatted: Font: (Default) Times New Roman

Data management

Personal data generated in the form of signed consent forms, personal mobile phone numbers, interviews, and/or focus group audio recordings, were stored following the 2018 Data Protection Act on a secure administrative database on a University of Oxford server.

Formatted: Heading 2, Line spacing: single

Results

Formatted: Heading 1, Line spacing: single

Pre-Trial Focus Group Discussions

Formatted: Heading 2, Line spacing: single, Border: Top: (No border), Bottom: (No border), Left: (No border), Right: (No border), Between : (No border)

Pre-trial focus group discussions (FGDs) were run in three groups of 12-13 participants in each trial site (37 people per trial site, 148 participants in total). In the pre-trial FGDs, themes identified for participant motivation were monetary benefit (63.5% of all participants), contributing to science/health (33.1%), having a phone (29.7%), electricity access (27.0%) (not a component of this study), the HumBug net (6.7%), and gaining personal knowledge (5.4%). Participants were typically motivated by multiple factors. As a participant in Bandundu describes ‘To do the job well, I have to be motivated, in the sense that I have to have the material for the job first, and then I have to be paid a good payment so that I can do a good job”. There was no significant difference in the number of motives each participant listed at the start of the study between Bandundu and Kinshasa ($p > 0.05$).

Statistical comparisons using Wilcoxon Rank Sum Tests showed that trial site participants in Kinshasa mentioned electricity significantly more as a reason to participate in the trial compared to Bandundu trial site participants ($W = 3774$, p-value < 0.001). Comparing treatment groups across both districts, electricity access was mentioned more frequently in the control groups compared to the incentive groups ($W = 2072$, p-value < 0.001). Between the treatment groups in Bandundu, electricity was more frequently mentioned by the control group (control n=6, incentive n=0; $W = 795.5$, p-value $= 0.012$) and personal knowledge (on topics such as mosquito control and malaria prevention) was more

commonly cited by the incentive group (control n=0, incentive n=4; W = 610.5, p-value = 0.042). In Kinshasa, the control group mentioned electricity (control n = 23, incentive n = 11; W = 906.5, p-value = 0.005) and contributing to science/health (control n = 15, incentive n = 6; W = 851, p-value = 0.022) significantly more than the incentive group. There were no other significant differences between districts, trial groups, or the trial groups within districts (p-value>0.05).

Demographic Survey

Formatted: Heading 2, Line spacing: single, Border: Top: (No border), Bottom: (No border), Left: (No border), Right: (No border), Between : (No border)

Demographic survey data were assessed to identify if they contributed to differences in recording ability between districts, treatment groups, and treatment groups within districts. There were significantly more adults in the homes of participants in the Kinshasa district sites compared to the Bandundu district sites (W = 1680, p-value = 0.017). Between the treatment groups in both districts, there were no significant differences in any demographic response variables. Within Kinshasa, there was significantly less water scarcity in the control group (29% experiencing water scarcity), and compared to the incentive group (45% experiencing water scarcity) (odds ratio = 0.09, p-value = 0.021) and there was a significant difference in the method of home lighting, with significantly more homes in the control group using a rechargeable flashlight, torch, or lantern (p-value = 0.016). Within Bandundu there were significantly more children in the incentive group compared to the control group (W = 675, p-value = 0.024), with one more child per house in the study on average. Additionally, there was a significant difference in toilet types between the Bandundu trial groups (p-value = 0.033). These were the only cases out of 88 comparisons that were significantly different between districts and trial groups (S3 Table).

Comparing Districts and Trial Groups

Formatted: Heading 2, Line spacing: single

Some participants decided to withdraw from the trial during its course and others failed to upload data, resulting in overall participation of 33 from the control group and 31 from the incentive group in the Kinshasa districts (n=64), and 33 from the control group and 35 from the incentive group in Bandundu districts (n=68), totalling 132 participants. The withdrawal rate from the study was 7.4% from the control sites and 10.4% from incentive sites across both districts. The reasons cited for formal withdrawal from the trial were lack of monetary benefit, unplanned move from the trial area, and loss of the smartphone. Comparisons in participation between trial districts using Wilcoxon Continuity tests showed that Bandundu participants uploaded data more weeks of the trial period (W = 1589, $p < 0.001$) and had more total uploads (W = 1825.5, p = 0.009), than participants in Kinshasa, shown in Figure 35.

Fig. 35: Comparison of a) the total number of uploads per participant in Bandundu and Kinshasa and b) the number of weeks that each participant was active in Bandundu and Kinshasa. Bandundu is shown in red, and Kinshasa is shown in blue. Boxes show the interquartile range (IQR), with whiskers showing the upper and lower 25% of the data, and points showing the outliers.

Comparing treatment groups across both districts there was no difference in participation between the control group and the incentive group for either total uploads (W = 2409.5, p-value = 0.88), or participation weeks (W = 2510.5, p-value= 0.79). Within Kinshasa there was no significant difference between treatment groups in either uploads (W = 418.5, p-value = 0.057) or weeks of activity (W = 428, p-value = 0.073). In Bandundu, there were significantly more total uploads in the control group compared to the incentive group (W = 845.5, p-value = 0.026) but no significant difference in the average number of active weeks of each participant (W = 777, p-value = 0.13) (Table 1). There were no significant relationships found between in the demographic variables that were captured in the demographic questionnaire survey and the number of uploads or weeks of activity.

*Table 1: Results of the Mann Whitney-U Tests for the trial group comparisons, with * to denote significance, p-value < 0.05 *, p-value<0.01 **.*

Comparison		Response Variable	Group	Mann-Witney U	Median	IQR	P-Value
District		Uploads	Kinshasa	W=1825.5	117.5	79.75-161.75	0.009486***
			Bandundu		160	107-186	
		Weeks	Kinshasa	W=1589	11	8-14.25	0.0002879***
			Bandundu		15	12-16	
Treatment		Uploads	Control	W=2409.5	136	73-186	0.8822
			Incentive		140	94.5-172.5	
		Weeks	Control	W = 2510.5	13	7-16	0.7852
			Incentive		13	10-15	
Bandundu	Treatment	Uploads	Control	W=845.5	180	122.25-193.5	0.02643*
			Incentive		149.5	91-177.75	
		Weeks	Control	W=777	16	12-16	0.1307
			Incentive		13.5	11.5-16	
Kinshasa	Treatment	Uploads	Control	W=418.5	99	62-164	0.05705
			Incentive		140	96.5-160	
		Weeks	Control	W=428	11	6-14	0.07298
			Incentive		12	10-14.5	

Formatted: Font: (Default) Times New Roman

Formatted: Font: (Default) Times New Roman

Formatted: Font: (Default) Times New Roman

Formatted: Font: (Default) Times New Roman

Comparison	Response Variable	Group	Mann-Witney U	Median	IQR	P-Value
District	Uploads	Kinshasa	W=1825.5	117.5	79.75-161.75	0.009486***
		Bandundu		160	107-186	
	Weeks	Kinshasa	W=1589	11	8-14.25	0.0002879***
		Bandundu		15	12-16	
Treatment	Uploads	Control	W=2409.5	136	73-186	0.8822
		Incentive		140	94.5-172.5	
	Weeks	Control	W = 2510.5	13	7-16	0.7852
		Incentive		13	10-15	
Bandundu Treatment	Uploads	Control	W=845.5	180	122.25-193.5	0.02643*
		Incentive		149.5	91-177.75	
	Weeks	Control	W=777	16	12-16	0.1307
		Incentive		13.5	11.5-16	
Kinshasa Treatment	Uploads	Control	W=418.5	99	62-164	0.05705
		Incentive		140	96.5-160	
	Weeks	Control	W=428	11	6-14	0.07298
		Incentive		12	10-14.5	

Activity and Effort over Time

Activity Persistence

The percentage of active participants was catalogued weekly to assess whether the monetary incentive affected persistence in data collection over the trial period (Fig. 46). At the start of the trial, the Kinshasa incentive group had the highest participation (87.1%), followed by the Bandundu incentive (83.3%), Bandundu control (80.5%), and Kinshasa control (70.3%). Participation was approximately stable for the first half of the trial before declining from week 8. At the end of the trial both the Kinshasa

Formatted: Font: (Default) Times New Roman

Formatted: Font: (Default) Times New Roman

Formatted: Font: (Default) Times New Roman

Formatted: Left

Formatted: Heading 2, Line spacing: single

control and Kinshasa incentive group had dropped to 30-35% participation, Bandundu incentive dropped to 50% participation, and Bandundu control maintained 77.7% participation.

Fig. 46: The percentage of participants that activated the MozzWear app in each trial group during each week in the trial. The coloured lines coordinate with trial groups, showing Bandundu control in orange, Bandundu incentive in green, Kinshasa control in pink, and Kinshasa incentive in blue. Lines were fitted using locally estimated scatterplot smoothing (LOESS), with the 95% confidence interval for each treatment group indicated in grey.

Data Collection Effort

Formatted: Heading 3, Left, Line spacing: single

Data collection effort over the trial showed that in the first week the Kinshasa incentive group had the highest average uploads per active participant (12.26) followed by the Bandundu control (11.69), Bandundu incentive (10.83), and Kinshasa control (10.54). Data collection effort declined at around week seven, and at the end of the trial, the upload effort dropped for all groups, finishing in descending order with Bandundu control (11.29), Bandundu incentive (10.39), Kinshasa incentive (10.8), and Kinshasa control (9.23). Over the course of the study, Bandundu had significantly more uploads per participant compared to Kinshasa (W = 1825.5, p-value = 0.009), and the Bandundu control group had higher data collection effort than the incentive group (W=845.5, p-value = 0.026). but there was no significant difference between the control and incentive groups overall or within Kinshasa. Figure 57 below shows the weekly comparisons in data collection effort over the course of the trial between the trial districts, treatment types, and treatments within districts. Bandundu had a trend of higher uploads in weeks 2-4, 8, and 11 (p-value < 0.1), and significantly higher uploads in weeks 9, 10, and 12-16 (p-value < 0.05) compared to the Kinshasa groups. Comparisons of the treatment groups showed that the control groups had higher participation than the incentive groups in the final three weeks of the trial (p-value <0.05). Within Bandundu, the control group had higher participation in weeks 14 and 16 of

the trial compared to the incentive group (p-value <0.05), and within Kinshasa, the control group had trends of lower participation than the incentive group in weeks 1 and 9 of the trial (p-value < 0.1)

Fig. 57: Week-to-week comparisons of collection effort between the trial districts, and incentive and control groups overall and within Kinshasa and Bandundu. The magnitude of the bars shows the difference in uploads per person from the first group in panel subtitle relative to the second. Non-significant differences are shown in grey; trends are shown in green (p-value < 0.1), significant results in yellow (p-value < 0.05), and highly significant differences are shown in red (p-value < 0.001). The whiskers around each bar show the standard error.

Post-Trial Focus Group Discussions

Formatted: Heading 2, Line spacing: single

The focus group discussions after the trial were attended by 27 participants from Kinshasa and 26 from Bandundu equating to ~35% of the original trial participants. In comparison to the 12 pre-trial FGDs, only 6 post-trial FGDs were run. Post-trial FGD attendance was lower in the Kinshasa incentive group compared to other groups (n = 8, p-value = 0.098, 95% CI: [0.0995, 1.2767] odds ratio: 0.368). All returning participants indicated that they had positive experiences with the HumBug sensor and a positive experience with the HumBug bed nets. When asked about the effect of the trial on their life, a respondent from the Bandundu incentive site said, "I was doing this work without someone to command me (...) it gave me the sense of responsibility."

There was a lower interest in future participation for control groups (n=12) compared to the incentive groups (n=25), and lower interest in future participation in Kinshasa (44.4% of respondents) compared to Bandundu (96.2% of respondents). Both district and trial group were significantly associated with participants interest in future participation (p-value < 0.001, 95% CI: [1.801, 5.468], odds ratio: 3.102), showing that in Bandundu the control group was 10% less likely to say they would participate in the

future than the Bandundu incentive group, and in Kinshasa the control group was 60% less likely to participate in the future compared to the Kinshasa incentive group.

When asked about challenges or things they wanted to change in the trial, electricity access was mentioned more frequently in the Kinshasa incentive group (W = 32, p-value = 0.007) and the Bandundu control group (W = 129, p-value = 0.008) compared to the other trial group in their respective districts. Financial compensation/increased compensation was mentioned by 17 respondents, but did not vary significantly between trial groups. When queried on how to improve the study, a respondent indicated a desire for more interaction and information, "When we send the data, on your part, we need a sign to know if the data has arrived or not (...) until the end, we had no communication."

Finally, only participants in the Kinshasa control group mentioned wanting to keep the smartphone (n=12). Despite not being discussed in other post-trial FGDs, phone retention was an issue at the end of the study. In total, there were 33 phones retained by participants across the districts, with a higher likelihood for the Kinshasa incentive group and for the Bandundu control group to retain phones, compared to the other trial group in their district (p-value=0.00516, 95% CI [1.567, 74.361], odds ratio: 9.177). Additionally, nine phones were reported as lost or stolen by the end of the trial period. As one participant phrased it from the control trial group in Kinshasa, “I want to be left with the phones because we will be laughed at (…) if you take them away from us, they will say that we participated in vain.”

Discussion

Formatted: Heading 1, Line spacing: single

Our results demonstrate that in the DRC there was no significant difference in the number of audio recordings uploaded or the number of weeks that participants were active between the groups receiving

financial incentives and the groups that did not. The previous HumBug study in Tanzania similarly showed no significant effect of incentives (monetary incentives, text message reminders, or their combination) on the total audio recordings uploaded ([34]). We found there was no significant effect of demographics such as age, sex, or income on the number of uploads or participation weeks, nor were there significant differences in these demographic traits between the districts, trial groups, or trial groups within districts. The only significant effect on the number of uploads and active weeks was study location which showed that participation was overall higher in Bandundu compared to Kinshasa, and a difference between the control and treatment group uploads in Bandundu due to a higher recording effort of the control group. This suggests that differences between the districts are not attributable to demographic effects, and instead, there may be other differences, such as intrinsic motivations or community leadership, resulting in higher numbers of uploads in Bandundu compared to the Kinshasa sites. These differences may be related to the proximity of the rural Kinshasa sites with the city of Kinshasa, though the access of participants to urban environments and differences in community cohesion were not specifically accounted for in our surveys.

Our study results also show that incentivisation did not significantly affect audio uploads or activity within Kinshasa and showed that the control group had more audio uploads in Bandundu compared to the incentive group. Previous studies have shown that if incentivised actions align with social norms, incentives can bolster desired behaviours ([543]), however, incentives can also weaken participants' intrinsic motivations—a phenomenon known as the 'crowding out' effect. This occurs when the anticipated intrinsic benefits of participation, such as the satisfaction of contributing, are diminished by the introduction of external incentives ([554]). The lack of response to incentives in our study suggests that incentives were neither supporting intrinsic motivations nor diminishing them. Notably, for logistical reasons this trial used individual health districts as trial groups. Differences in response to incentives may be more strongly attributed to local culture and attitudes than treatment group. In

the future, a randomised control trial that assesses participants' existing motivations could more effectively capture variations both among and within localities engaged in data collection. Additionally, further work is needed to understand what the tipping points are for incentives to significantly motivate participation, and what behaviours may either be unaffected by incentivisation or diminished by it.

Although incentives did not affect the overall number of data uploads across districts, there were differences in the persistence of data collection participation and effort. The participation persistence of the trial groups showed that the Bandundu control group had approximately the same amount of participation from the start to the end of the trial, but that the other three trial groups experienced significant declines in the latter half of the trial. A review paper of citizen science studies found that studies that provided long term training and contact with organisers were the most successful at engaging participants ([565]). The declines in robust participation towards the end of the trial in our study may be attributed to the lack of engagement with participants that was necessary to test the usability of the data collection methodology with low intervention. The low contact resulted in several post-trial focus group respondents indicating they would have liked more contact, despite all participants saying the sensor and application were easy to use. Although participants had positive attitudes regarding the data collection and responsibility (supporting results from other studies ([576])) access to data and collaborators may further improve participants’ feelings about their participation and their contributions ([587]).

Participant-researcher contact may be why our previous trial in Tanzania found that text reminders, both alone and paired with an incentive, positively affected persistence in data collection, despite the monetary incentive alone not showing a significant effect in that study system ([34]). The declines in participation persistence and effort seen in this study support this previous finding that incentives alone

are not enough to motivate participation. The tipping point in the middle of the study where participation dropped suggests that participants do not need further encouragement or motivation in the short term to collect Humbug data. In longer term contexts however, consistent contact and communication may be necessary to continue to motivate participants, and we have found that monetary incentives do not accomplish this, and in fact appear to only increase participation in the early phases of the study.

The differences in upload effort between our trial groups did not follow patterns relating to incentivisation. The data collection effort in Bandundu shows that the control group performed similarly to the incentive group for most of the trial, with the control group performing significantly better in the final couple of weeks. This appears to be due to a continued high level of data collection persistence and effort on the part of the Bandundu control group, which seems to be attributable to some intrinsic difference in this site compared to the other sites. In Kinshasa, incentivisation improved data collection efforts in the early parts of the trial compared to the control group, suggesting that at the time when participants were overall the most active, the incentive did improve the completeness of data collection efforts. However, this higher level of data collection effort by Kinshasa's incentive group dropped approximately halfway through the trial, after which there was not a significant difference between the control and incentive groups. This mirrors the results for participant activity, which showed that participation significantly dropped off halfway through. The decline in data collection efforts shows that even those who are continuing to participate are not collecting data for the entire time period (6 pm to 6 am) as requested in the data collection methodology. This may be in part due to the design of our incentives, which were given regardless of activity and were not scaled for completeness of participation. This result shows that despite incentivisation initially appearing to motivate complete data collection in one of our districts, the diminishing interest and effort over time are not overcome by incentivisation.

Our post-trial FGDs gave insight into issues surrounding incentives, including compensation and technology. Over half of the participants in the trial did not return for the post-trial FGDs. The reasons cited by the DRC team were a desire to keep the smartphone and the lack of payment at the control sites, though there were more post-trial FGD attendees from the control groups than the incentive groups. This compensation issue was emphasised by a refusal to return the phones by some participants in trial groups in both districts. The Bandundu control group exhibited higher phone retention compared to the Bandundu incentive group, and the Kinshasa incentive group showed higher phone retention in contrast to the Kinshasa control group. There was no significant difference in the number of participants that named phone retention as a motive for participation between the districts, the trial groups, or between the trial groups within districts, showing that previously held values were not associated with phone retention. Instead, the pattern of phone retention mirrored the activity levels of each trial group, perhaps indicating that the more active participants felt a stronger entitlement to keeping the phones. However, phone retention was related to the relative effort in each district, rather than the overall effort. For example, the Bandundu incentive and the Kinshasa incentive had similar activity levels and total uploads, yet the Kinshasa incentive group retained twice the number of phones. As far as we are aware, participants in each district and trial group did not know the participation rates of the other groups, and therefore, did not know their relative participation. This indicates that individuals' participation might be influenced by their perception of effort.

The phone retention and refusal to return at the end of the study is an example of how citizen science projects can deviate from planned outcomes. In this context, participants effectively provided themselves with an incentive (or supplementary incentive) by refusing to return phones, despite study agreements and information about compensation ahead of the trial. A study on citizen science with specially designed video games found that game design can diminish citizen science outcomes and

enhance the odds of cheating and cutting corners, depending on the ability to do so ([598]). It is important to consider how incentive application design therefore may influence study outcomes and participation. The incentive group in this case received payment regardless of participation, which may have opened this study system to cutting corners (e.g., reduced activity and upload effort at the end of the study period). As an alternative, incentives given in response to data collection activity close this loophole and effectively encourage data collection persistence. Additionally, the post-trial survey in this study was not incentivised, and therefore participants gained more from refusing to return phones than they did from attending. Balancing incentive value with requests and alternatives is necessary for incentives to operate as intended, and as seen in this study, some individuals will not follow prescriptive actions if they feel they can benefit in other ways.

Our post-trial FGDs revealed that despite there not being a significant or differential response to incentivisation, there were significant contrasting attitudes between the study groups in Bandundu and those in Kinshasa. Bandundu exhibited a significantly higher overall participation rate in FGDs compared to Kinshasa, and a greater proportion of Bandundu participants expressed willingness to partake in the trial again, irrespective of incentivisation (25 out of 26 post-trial FGD attendees). Out of 27 post-trial FGD attendees from Kinshasa, only 12 indicated a willingness to participate again. Additionally, there appear to be differences in values between the two districts. Among the 16 participants who identified knowledge as their motive for future participation, only one was from Kinshasa. Conversely, among Kinshasa participants who expressed willingness to participate again, the majority cited incentives as their driving factor. Further research is needed to understand how community and culture interact with incentive application, but in this case, despite differing motivations for participation, incentives did not have any significant effects on overall participation in the study.

The biggest issues cited by participants who took part in the trial were access to electricity to charge the smartphone sensor and internet connection. Interestingly, the groups that mentioned access to electricity significantly more were also the groups that performed best for data collection activity and effort in each district. It could be that the practicality of it was more apparent to the groups that put more effort in, and practical difficulties may be part of the reason people felt they should have been compensated better for the study. Of the incentive group participants who attended the focus group, over half said they felt they had not been given enough money for the study (14/22). This dissatisfaction may have been due to the unexpected cost of transferring money, which reduced the originally stated compensation (from $10 to $9). In pre-trial FGDs, there were only six cases when participants who cited money as their motivation asked for a monetary value at or below the incentive value given in the study, suggesting that the incentive potentially fell below common expectations.

Despite the sentiment that the money was not enough, incentive group participants were more likely to say they would participate in the future than control group participants, and although bed nets were one of the least cited reasons for participating in the study, almost all participants mentioned the bed nets as a benefit they experienced in the trial, perhaps improving their desire to continue participation. Considering that all phones were turned on and collected data at least once during the study period, it appears useability was not a limiting factor of the study, and indeed all participants apart from one said that they felt the MozzWear app was easy to use. Due to the lack of practical issues with the study, our results highlight the importance of scaling expectations of citizen science and matching the value of incentives with the effort and length of trials to improve participation and satisfaction.

Our findings show that mobile applications using hardware on budget smartphones can generate useful data with limited interventions and suggests that mobile application development should be more widely considered in citizen science data collection efforts. Future research on the role of

Formatted: Font: (Default) Times New Roman, 12 pt, Not Bold, Font color: Auto

Formatted: Justified, Space Before: 0 pt, After: 0 pt, Line spacing: Double, Font Alignment: Auto, Pattern: Clear

incentivization for data collection that utilizes technology should consider how to further disambiguate the inherent benefits in participation related to technological access, such as social status or monetary benefit from retaining equipment. We suggest that with increased mobile phone ownership, this complexity may be overcome by utilizing current phone owners, rather than providing participants with hardware. Additionally, as mobile phone use becomes more popular, opportunities for large scale studies will support more complex statistical approaches to account for the complex structures that underpin intrinsic motivators and responses to extrinsic motivators, such as proximity to urban areas which are potentially obscured by the inferential statistical methods used in this study.

Formatted: Font: (Default) Times New Roman

Formatted: Font: (Default) Times New Roman

Formatted: Font: (Default) Times New Roman, 12 pt, Not Bold, Font color: Auto

Formatted: Font: (Default) Times New Roman

Conclusions

Formatted: Heading 1, Line spacing: single

Results of this study show that in this study system incentives did not have significant effects on data collection activity throughout the trial but did have some effect on data collection persistence and effort, improving data collection effort during portions of the trial in one of our study districts. Our results indicate that community differences are what drive the trends in participation in this study system, and that more information is needed ahead of trials to assess how community differences may impact participation in citizen science data collection. Participant engagement with researchers and study organisers appears to improve data collection, particularly in longer studies. Communication with participants is important for appropriately scaling incentives to account for citizen data collection effort. Systems should be designed appropriately to maximise the alignment of incentives with active data collection. Overall, the incentives tested do not appear to significantly improve data collection in this study system, and in this case, increased engagement during data collection may have improved outcomes. With technology becoming an increasingly used tool in citizen science, its application should be considered in reference to existing infrastructure. Consideration should be made for the

value that the technology represents in reference to the overall value of the incentive being offered to avoid issues with data sensor retention in response to changing attitudes towards incentive value.

Acknowledgements

Thank you to the DRC team at Kinshasa School of Public Health and University of Bandundu and to Paul Mansiangi, who has since passed away, for organising and co-ordinating the study. We are grateful to the community leaders, health zone officials, and all the participants from Trois Rivières, Caravane, Bu and Mikondo for their support and participation in the study.

~~Ethics Approval and Consent~~

~~The University of Oxford have sponsored the study. This study was reviewed by the Oxford Tropical Research Ethics Committee (OxTREC) and approved by the Kinshasa School of Public Health. Informed written consent was obtained from all interviewees prior to the focus group discussions and the quantitative feedback survey. Consent forms signed by all the participants included the release of summary findings and details of individual responses from this study. Potential participants were informed of the voluntary nature of the study and had at least 24 hours to consider taking part. Efforts were made to create a safe place for sharing experiences during the focus group discussions.~~

References

1. GSMA. GSMA | The Mobile Economy - Sub-Saharan Africa 2017 - GSMA Sub Saharan Africa [Internet]. 2017 [cited 2023 Aug 28]. Available from: https://www.gsma.com/subsaharanafrica/resources/mobile-economy-2017-sub-saharan-africa-2017
2. GSMA. GSMA | The State of Mobile Internet Connectivity Report 2019 | Mobile for Development [Internet]. 2019 [cited 2023 Aug 28]. Available from:

Formatted: Heading 1, Line spacing: single, Border: Top: (No border), Bottom: (No border), Left: (No border), Right: (No border), Between : (No border)

Formatted: Font: (Default) Times New Roman

Formatted: Heading 1, Left, Line spacing: single

Formatted: Font: (Default) Times New Roman, English (United Kingdom)

Formatted: Font: (Default) Times New Roman, Not Bold

Formatted: Font: (Default) Times New Roman

Formatted: Heading 1, Line spacing: single

https://www.gsma.com/mobilefordevelopment/resources/the-state-of-mobile-internet-connectivity-report-2019/

3. ITU Hub. Measuring digital development: Facts & figures 2019 - ITU Hub [Internet]. 2019 [cited 2023 Aug 28]. Available from: https://www.itu.int/hub/2020/05/measuring-digital-development-facts-figures-2019/
4. Baxter C, Carroll JA, Keogh B, Vandelanotte C. Assessment of Mobile Health Apps Using Built-In Smartphone Sensors for Diagnosis and Treatment: Systematic Survey of Apps Listed in International Curated Health App Libraries. JMIR Mhealth Uhealth. 2020 Feb 3;8(2):e16741.
5. Kamel Boulos MN, Brewer AC, Karimkhani C, Buller DB, Dellavalle RP. Mobile medical and health apps: state of the art, concerns, regulatory control and certification. Online J Public Health Inform. 2014 Feb 5;5(3).
6. Lee SH, Nurmatov UB, Nwaru BI, Mukherjee M, Grant L, Pagliari C. Effectiveness of mHealth interventions for maternal, newborn and child health in low– and middle–income countries: Systematic review and meta–analysis. J Glob Health [Internet]. 2016 [cited 2023 Aug 28];6(1). Available from: /pmc/articles/PMC4643860/
7. Lee Ventola C. Mobile Devices and Apps for Health Care Professionals: Uses and Benefits. Pharmacy and Therapeutics [Internet]. 2014 [cited 2023 Aug 28];39(5):356. Available from: /pmc/articles/PMC4029126/
8. Barrington J, Wereko-Brobby O, Ward P, Mwafongo W, Kungulwe S. SMS for Life: a pilot project to improve anti-malarial drug supply management in rural Tanzania using standard technology. Malar J. 2010 Dec 27;9(1):298.
9. Davis RG, Kamanga A, Castillo-Salgado C, Chime N, Mharakurwa S, Shiff C. Early detection of malaria foci for targeted interventions in endemic southern Zambia. Malar J [Internet]. 2011 Sep 12 [cited 2023 Aug 28];10(1):1–12. Available from: https://malariajournal.biomedcentral.com/articles/10.1186/1475-2875-10-260
10. Francis F, Ishengoma DS, Mmbando BP, Rutta ASM, Malecela MN, Mayala B, et al. Deployment and use of mobile phone technology for real-time reporting of fever cases and malaria treatment failure in

areas of declining malaria transmission in Muheza district north-eastern Tanzania. Malar J [Internet]. 2017 Aug 1 [cited 2023 Aug 28];16(1). Available from: https://pubmed.ncbi.nlm.nih.gov/28764792/

11. Gibson DG, Ochieng B, Kagucia EW, Were J, Hayford K, Moulton LH, et al. Mobile phone-delivered reminders and incentives to improve childhood immunisation coverage and timeliness in Kenya (M-SIMU): a cluster randomised controlled trial. Lancet Glob Health [Internet]. 2017 Apr 1 [cited 2023 Aug 28];5(4):e428–38. Available from: https://pubmed.ncbi.nlm.nih.gov/28288747/

12. Kamanga A, Moono P, Stresman G, Mharakurwa S, Shiff C. Rural health centres, communities and malaria case detection in Zambia using mobile telephones: A means to detect potential reservoirs of infection in unstable transmission conditions. Malar J [Internet]. 2010 Apr 15 [cited 2023 Aug 28];9(1):1–7. Available from: https://malariajournal.biomedcentral.com/articles/10.1186/1475-2875-9-96

13. Kaunda-Khangamwa BN, Steinhardt LC, Rowe AK, Gumbo A, Moyo D, Nsona H, et al. The effect of mobile phone text message reminders on health workers' adherence to case management guidelines for malaria and other diseases in Malawi: lessons from qualitative data from a cluster-randomized trial. Malar J [Internet]. 2018 Dec 19 [cited 2023 Aug 28];17(1). Available from: https://pubmed.ncbi.nlm.nih.gov/30567603/

14. Meankaew P, Kaewkungwal J, Khamsiriwatchara A, Khunthong P, Singhasivanon P, Satimai W. Application of mobile-technology for disease and treatment monitoring of malaria in the "Better Border Healthcare Programme." Malar J [Internet]. 2010 Aug 19 [cited 2023 Aug 28];9(1):1–14. Available from: https://link.springer.com/articles/10.1186/1475-2875-9-237

15. Wakadha H, Chandir S, Were EV, Rubin A, Obor D, Levine OS, et al. The feasibility of using mobile-phone based SMS reminders and conditional cash transfers to improve timely immunization in rural Kenya. Vaccine [Internet]. 2013 Jan 30 [cited 2023 Aug 28];31(6):987–93. Available from: https://pubmed.ncbi.nlm.nih.gov/23246258/

16. Roche J, Bell L, Galvão C, Golumbic YN, Kloetzer L, Knoben N, et al. Citizen Science, Education, and Learning: Challenges and Opportunities. Frontiers in Sociology. 2020 Dec 2;5:613814.

17. Conrad CC, Hilchey KG. A review of citizen science and community-based environmental monitoring: Issues and opportunities. Environ Monit Assess [Internet]. 2011 May 17 [cited 2023 Oct 2];176(1–4):273–91. Available from: https://link.springer.com/article/10.1007/s10661-010-1582-5

18. Den Broeder L, Devilee J, Van Oers H, Schuit AJ, Wagemakers A. Citizen Science for public health. Health Promot Int [Internet]. 2018 Jun 1 [cited 2023 Oct 2];33(3):505–14. Available from: https://dx.doi.org/10.1093/heapro/daw086

19. Di Domenico SI, Ryan RM. The emerging neuroscience of intrinsic motivation: A new frontier in self-determination research. Front Hum Neurosci. 2017 Mar 24;11:247005.

20. Hennessey B, Moran S, Altringer B, Amabile TM. Extrinsic and Intrinsic Motivation. Wiley Encyclopedia of Management. 2015 Jan 21;1–4.

21. Ryan RM, Deci EL. Intrinsic and extrinsic motivation from a self-determination theory perspective: Definitions, theory, practices, and future directions. Contemp Educ Psychol. 2020 Apr 1;61.

22. Aceves-Bueno E, Adeleye AS, Bradley D, Tyler Brandt W, Callery P, Feraud M, et al. Citizen Science as an Approach for Overcoming Insufficient Monitoring and Inadequate Stakeholder Buy-in in Adaptive Management: Criteria and Evidence. Ecosystems [Internet]. 2015 Apr 1 [cited 2023 Oct 2];18(3):493–506. Available from: https://link.springer.com/article/10.1007/s10021-015-9842-4

23. Cetas ER, Yasué M. A systematic review of motivational values and conservation success in and around protected areas. Conserv Biol [Internet]. 2017 Feb 1 [cited 2023 Aug 28];31(1):203–12. Available from: https://pubmed.ncbi.nlm.nih.gov/27254293/

24. Lester RT, Ritvo P, Mills EJ, Kariri A, Karanja S, Chung MH, et al. Effects of a mobile phone short message service on antiretroviral treatment adherence in Kenya (WelTel Kenya1): a randomised trial. Lancet [Internet]. 2010 Nov 27 [cited 2023 Aug 28];376(9755):1838–45. Available from: https://pubmed.ncbi.nlm.nih.gov/21071074/

25. Ngabo F, Nguimfack J, Nwaigwe F, Mugeni C, Muhoza D, Wilson DR, et al. Designing and Implementing an Innovative SMS-based alert system (RapidSMS-MCH) to monitor pregnancy and reduce maternal and child deaths in Rwanda. Pan Afr Med J [Internet]. 2012 [cited 2023 Aug 28];13. Available from: /pmc/articles/PMC3542808/

26. Richter A, Comay O, Svenningsen CS, Larsen JC, Hecker S, Tøttrup AP, et al. Motivation and support services in citizen science insect monitoring: A cross-country study. Biol Conserv. 2021 Nov 1;263.

27. Tomlinson M, Solomon W, Singh Y, Doherty T, Chopra M, Ijumba P, et al. The use of mobile phones as a data collection tool: a report from a household survey in South Africa. BMC Med Inform Decis Mak [Internet]. 2009 [cited 2023 Aug 28];9(1). Available from: https://pubmed.ncbi.nlm.nih.gov/20030813/

28. Bell S, Marzano M, Cent J, Kobierska H, Podjed D, Vandzinskaite D, et al. What counts? Volunteers and their organisations in the recording and monitoring of biodiversity. Biodivers Conserv. 2008 Dec 26;17(14):3443–54.

29. Finkelstien MA. Intrinsic vs Extrinsic Motivational Orientations and the Volunteer Process. Pers Individ Dif [Internet]. 2009 Jan 1 [cited 2023 Aug 28];46(5–6):653. Available from: https://digitalcommons.usf.edu/psy_facpub/770

30. Hobbs SJ, White PCL. Motivations and barriers in relation to community participation in biodiversity recording. J Nat Conserv [Internet]. 2012 Dec [cited 2023 Aug 28];20(6):364–73. Available from: https://pure.york.ac.uk/portal/en/publications/motivations-and-barriers-in-relation-to-community-participation-i

31. Jordan Raddick M, Bracey G, Gay PL, Lintott CJ, Cardamone C, Murray P, et al. Galaxy zoo: Motivations of citizen scientists. Astronomy Education Review. 2013;12(1).

32. Cerasoli CP, Nicklin JM, Ford MT. Intrinsic motivation and extrinsic incentives jointly predict performance: A 40-year meta-analysis. Psychol Bull. 2014;140(4):980–1008.

33. Patall EA, Cooper H, Robinson JC. The effects of choice on intrinsic motivation and related outcomes: a meta-analysis of research findings. Psychol Bull [Internet]. 2008 Mar [cited 2023 Aug 28];134(2):270–300. Available from: https://pubmed.ncbi.nlm.nih.gov/18298272/

34. Dam R, Mponzi W, Msaky D, Mwandyala T, Kaindoa EW, Sinka ME, et al. What incentives encourage local communities to collect and upload mosquito sound data by using smartphones? A mixed methods study in Tanzania. Glob Health Res Policy. 2023 Dec 1;8(1).

35. Sinka ME, Zilli D, Li Y, Kiskin I, Msaky D, Kihonda J, et al. HumBug – An Acoustic Mosquito Monitoring Tool for use on budget smartphones. Methods Ecol Evol [Internet]. 2021 Oct 1 [cited 2023

Aug 28];12(10):1848–59. Available from: https://onlinelibrary.wiley.com/doi/full/10.1111/2041-210X.13663

36. Kiskin I, Zilli D, Li Y, Sinka M, Willis K, Roberts S. Bioacoustic detection with wavelet-conditioned convolutional neural networks. Neural Comput Appl. 2020 Feb 1;32(4):915–27.

37. Li Y, Kiskin I, Zilli D, Sinka M, Chan H, Willis K, et al. Cost-sensitive detection with variational autoencoders for environmental acoustic sensing. 2017 Dec 6 [cited 2023 Dec 7]; Available from: http://arxiv.org/abs/1712.02488

38. Kiskin I, Orozco BP, Windebank T, Zilli D, Sinka M, Willis K, et al. Mosquito Detection with Neural Networks: The Buzz of Deep Learning. 2017 May 15 [cited 2023 Dec 7]; Available from: http://arxiv.org/abs/1705.05180

39. Li Y, Zilli D, Chan H, Kiskin I, Sinka M, Roberts S, et al. Mosquito detection with low-cost smartphones: data acquisition for malaria research. 2017 Nov 16 [cited 2023 Dec 7]; Available from: http://arxiv.org/abs/1711.06346

40. Kiskin I, Cobb AD, Sinka M, Willis K, Roberts SJ. Automatic Acoustic Mosquito Tagging with Bayesian Neural Networks. Lecture Notes in Computer Science (including subseries Lecture Notes in Artificial Intelligence and Lecture Notes in Bioinformatics). 2021;12978 LNAI:351–66.

41. Kiskin I, Cobb AD, Wang L, Roberts S. Humbug Zooniverse: A Crowd-Sourced Acoustic Mosquito Dataset. ICASSP, IEEE International Conference on Acoustics, Speech and Signal Processing - Proceedings. 2020 May 1;2020-May:916–20.

42. Li Y, Kiskin I, Sinka M, Zilli D, Chan H, Herreros-Moya E, et al. Fast mosquito acoustic detection with field cup recordings: an initial investigation. dcase.community [Internet]. 2018 [cited 2024 Aug 16]; Available from: https://dcase.community/documents/workshop2018/proceedings/DCASE2018Workshop_Li_136.pdf

43. Centers for Disease Control and Prevention. CDC in the Democratic Republic of the Congo | Global Health | CDC [Internet]. 2023 [cited 2023 Aug 28]. Available from: https://www.cdc.gov/globalhealth/countries/drc/default.htm

44. World Health Organisation. World malaria report 2022 [Internet]. 2022 [cited 2023 Aug 28]. Available from: https://www.who.int/teams/global-malaria-programme/reports/world-malaria-report-2022

45. SimpleMaps.com. "Democratic Republic of the Congo (DRC) GIS Data." *SimpleMaps.com*, 2024, https://simplemaps.com/gis/country/cd#admin1. Accessed 16 Oct. 2024. Licensed under Creative Commons Attribution License. https://creativecommons.org/licenses/by/4.0/

46. Center for International Earth Science Information Network (CIESIN), Columbia University, Ministère de la Santé Publique, Hygiène et Prévention, Democratic Republic of the Congo, and GRID3. (2025). *GRID3 COD - Health Areas v4.0: Kinshasa health areas (GeoPackage)*. New York: Columbia University. https://data.humdata.org/dataset/grid3-cod-health-areas-v4-0. Accessed April 3, 2025. Licensed under Creative Commons Attribution License. https://creativecommons.org/licenses/by/4.0/~~Humanitarian Data Exchange. DRC health data. Humanitarian Data Exchange; 2024. Available from: https://data.humdata.org/dataset/drc-health-data. Accessed on: 14 Oct 2024.~~

Formatted: Font: (Default) Times New Roman

Formatted: Font: (Default) Times New Roman

Formatted: Font: (Default) Times New Roman

Formatted: Font: (Default) Times New Roman

47. Center for International Earth Science Information Network (CIESIN), Columbia University, Ministère de la Santé Publique, Hygiène et Prévention, Democratic Republic of the Congo, and GRID3. (2025). *GRID3 COD - Health Areas v4.0: Kwilu health areas (GeoPackage)*. New York: Columbia University. https://data.humdata.org/dataset/grid3-cod-health-areas-v4-0. Accessed April 3, 2025. Licensed under Creative Commons Attribution License. https://creativecommons.org/licenses/by/4.0/

Formatted: Font: (Default) Times New Roman

Formatted: Font: (Default) Times New Roman

Formatted: Font: (Default) Times New Roman

Formatted: Font: (Default) Times New Roman

48~~7~~. QGIS Development Team. QGIS [3.0.0]. Open Source Geospatial Foundation; 2018. Available from: https://qgis.org/en/site/.

49~~8~~. Saldaña Johnny. The Coding Manual for Qualitative Researchers. 2021;1–440.

50~~49~~. R Core Team. R: A Language and Environment for Statistical Computing_. 2023; R Foundation for Statistical Computing, Vienna, Austria. <https://www.R-project.org/>.

51~~0~~. Kruskal WH, Wallis WA. Use of ranks in one-criterion variance analysis. J Am Stat Assoc. 1952;47:583-621. Available from: http://dx.doi.org/10.1080/01621459.1952.10483441.

Formatted: Font: (Default) Times New Roman

Formatted: Font: (Default) Times New Roman

52~~1~~. Dinno A. dunn.test: Dunn's Test of Multiple Comparisons Using Rank Sums. R package. 2024. Available from: <https://CRAN.R-project.org/package=dunn.test>.

53~~2~~. Wickham H. ggpolt2 Elegant Graphics for Data Analysis. Use R! series. 2016;211.

543. Kerr JM, Bum T, Lapinski MK, Liu RW, Lu Z, Zhao J. The effects of social norms on motivation crowding: experimental evidence from the Tibetan Plateau. Int J Commons [Internet]. 2019 [cited 2023 Aug 28];13(1):430–54. Available from: https://thecommonsjournal.org/articles/10.18352/ijc.882

554. Rode J, Gómez-Baggethun E, Krause T. Motivation crowding by economic incentives in conservation policy: A review of the empirical evidence. Ecological Economics. 2015 Sep 1;117:270–82.

565. Aceves-Bueno E, Adeleye AS, Feraud M, Huang Y, Tao M, Yang Y, et al. The Accuracy of Citizen Science Data: A Quantitative Review. Bulletin of the Ecological Society of America. 2017 Oct;98(4):278–90.

576. Peter M, Diekötter T, Kremer K. Participant Outcomes of Biodiversity Citizen Science Projects: A Systematic Literature Review. Sustainability 2019, Vol 11, Page 2780 [Internet]. 2019 May 15 [cited 2023 Oct 1];11(10):2780. Available from: https://www.mdpi.com/2071-1050/11/10/2780/htm

587. Ganzevoort W, van den Born RJG, Halffman W, Turnhout S. Sharing biodiversity data: citizen scientists' concerns and motivations. Biodivers Conserv [Internet]. 2017 Nov 1 [cited 2023 Oct 1];26(12):2821–37. Available from: https://link.springer.com/article/10.1007/s10531-017-1391-z

598. Prestopnik N, Crowston K. Purposeful gaming & socio-computational systems. In: Proceedings of the 17th ACM international conference on Supporting group work. New York, NY, USA: ACM; 2012. p. 75–84.

Supplementary Information

Formatted: Heading 1, Line spacing: single

S1 Appendix: The DHS Demographic Survey Administered to Participants

S2 Appendix: The focus group discussion questions administered pre-trial and post-trial.

S3 Table: A table of results from comparisons of the demographic survey between study districts, trial groups overall, and trial groups within each district.

Dear Dr. da Luz,

Thank you for the opportunity to submit a revised draft of our manuscript titled "*What incentives encourage local communities to collect and upload mosquito sound data using smartphones? A case study in the Democratic Republic of the Congo*" to *PLOS One*. We appreciate the time you and the reviewers have taken to provide valuable feedback and insightful comments. We have made changes to incorporate most of the suggestions provided by reviewers and they are highlighted through the manuscript. Please find below the point-by-point responses and edits made to the paper in line with feedback.

Journal Requirements:

Comment 1*: Please ensure that your manuscript meets PLOS ONE's style requirements, including those for file naming.*

Response: The manuscript has been amended to address the style and naming requirements.

Comment 2: *Please note that funding information should not appear in any section or other areas of your manuscript. We will only publish funding information present in the Funding Statement section of the online submission form. Please remove any funding-related text from the manuscript.*

Response: The manuscript has been amended to remove funding information from the manuscript.

Comment 3: *When completing the data availability statement of the submission form, you indicated that you will make your data available on acceptance. We strongly recommend all authors decide on a data sharing plan before acceptance, as the process can be lengthy and hold up publication timelines. Please note that, though access restrictions are acceptable now, your entire data will need to be made freely accessible if your manuscript is accepted for publication. This policy applies to all data except where public deposition would breach compliance with the protocol approved by your research ethics board. If you are unable to adhere to our open data policy, please kindly revise your statement to explain your reasoning and we will seek the editor's input on an exemption. Please be assured that, once you have provided your new statement, the assessment of your exemption will not hold up the peer review process.*

Response: All the raw data from our study is now publicly available at 10.6084/m9.figshare.27332124

Comment 4*: We note that Figure 2 in your submission contain map/satellite images which may be copyrighted. All PLOS content is published under the Creative Commons Attribution License (CC BY*

4.0), which means that the manuscript, images, and Supporting Information files will be freely available online, and any third party is permitted to access, download, copy, distribute, and use these materials in any way, even commercially, with proper attribution. For these reasons, we cannot publish previously copyrighted maps or satellite images created using proprietary data, such as Google software (Google Maps, Street View, and Earth). For more information, see our copyright guidelines: http://journals.plos.org/plosone/s/licenses-and-copyright.

Response: To comply with the rules regarding CC BY 4.0 the original data showing the health districts of the DRC from the Humanitarian Data Exchange (DRC health Data) was removed and replaced using Creative Commons Attribution Licensed data. The Democratic Republic of the Congo (DRC) GIS data from simplemaps, the GRID3 COD - Health Areas v4.0: Kwilu health areas (GeoPackage), and the GRID3 COD - Health Areas v4.0: Kinshasa health area (GeoPackage) are all accessible under existing CC BY 4.0 licenses. This change in data used in the map of the DRC has also been reflected in the addition of two references for the GeoPackages used, along with the statement of origin in the caption on Figure 2:

Center for International Earth Science Information Network (CIESIN), Columbia University, Ministère de la Santé Publique, Hygiène et Prévention, Democratic Republic of the Congo, and GRID3. (2025). GRID3 COD - Health Areas v4.0: Kinshasa health areas (GeoPackage). New York: Columbia University. https://data.humdata.org/dataset/grid3-cod-health-areas-v4-0. Accessed April 3, 2025.

Center for International Earth Science Information Network (CIESIN), Columbia University, Ministère de la Santé Publique, Hygiène et Prévention, Democratic Republic of the Congo, and GRID3. (2025). GRID3 COD - Health Areas v4.0: Kwilu health areas (GeoPackage). New York: Columbia University. https://data.humdata.org/dataset/grid3-cod-health-areas-v4-0. Accessed April 3, 2025.

Comment 5: *We note that Figure 3 and 4 includes an image of a participant in the study.*

Response: These images have been removed from the manuscript.

Comment 6. *Please review your reference list to ensure that it is complete and correct. If you have cited papers that have been retracted, please include the rationale for doing so in the manuscript text or remove these references and replace them with relevant current references. Any changes to the reference list should be mentioned in the rebuttal letter that accompanies your revised manuscript. If you need to cite a retracted article, indicate the article's retracted status in the References list and also include a citation and full reference for the retraction notice.*

Response: The citation list has been reviewed and there were no instances of retracted papers. The reference list was amended with the GeoPackage references for the Kwilu and Kinshasa health districts (shown above), and the references were renumbered to reflect these additions.

Comments from Reviewer 1

Major comments

Comment 1: *Lines 79-85: the introduction references the Tanzania study but does not clearly position this manuscript as a follow-up. It could strengthen the manuscript to explicitly state how this work builds on or how it differs from the Tanzania findings. It is recommended that the authors add a sentence explicitly linking the gaps identified in the Tanzania study (for instance, rural-only context) to the objectives of this work.*

Response: This study is named directly as a second trial of the mosquito detection application with monetary incentives in line 187. Verbiage specifying the research's focus on the use of monetary extrinsic factors, and how the study builds on the study in Tanzania has been added in lines 205-206, stating: 'This study builds upon our previous work in Tanzania to fill gaps in understanding how monetary incentives alone influence the consistency and quality of audio data collection.'

Comment 2: *Regarding the Focus Group Insights, lines 247-253, the discussion of Focus Group Discussions (FGD) results is useful but could benefit from additional depth. For example, higher persistence in Bandundu could be contextualized using participant quotes. A suggestion would be that the authors add representative quotes from FGDs to illustrate key themes and link them to the observed participation trends.*

Response: Due to the lack of significant differences in pre-trial FGD responses between Kinshasa and Bandundu it does not feel appropriate to use a FGD response in this section of the results to highlight differences in the groups that were seen later in the trial (such as in the post-trial FGDs). Instead, a quote from a participant listing multiple motivation factors has been added in lines 373-377 to demonstrate how complex participant motivations were prior to trial participation.

Comment 3: *The study's inclusion of Bandundu (rural) and Kinshasa (urban) appear to be an opportunity to explore how rural-urban differences influence participation. However, the manuscript does not fully address these dynamics or their potential implications. In lines 401-402, the authors note that differences in participation are not attributable to demographic factors but do not explore other potential influences, such as rural-urban dynamics that may be marked in the two setting of the study. This section could be expanded on to discuss e.g. infrastructure, community cohesion, and leadership differences between Bandundu and Kinshasa.*

Response: This study was not aimed at specifically addressing urban versus rural factors for responses to incentivization, and the Kinshasa study sites were not located in the city but in rural parts of the

health district (referred to as rural Kinshasa when first introduced in the manuscript, line 188). However, we acknowledge the potential role of proximity to urban areas and have now addressed this in the manuscript discussion lines 547-549, reading: 'These differences may be related to the proximity of the rural Kinshasa sites with the city of Kinshasa, though the access of participants to urban environments and differences in community cohesion were not specifically accounted for in our surveys.'

Minor comments:

Comment 4: *Line 46: replace "effecting sub-Saharan Africa" with "affecting sub-Saharan Africa."*

Response: This has now been amended in the manuscript.

Comment 5: *Discussion: the authors acknowledge that the findings are context-specific but do not discuss how they might be generalised to other settings. The paper could benefit from expanding the discussion to include how differences in infrastructure, culture, or community norms might affect the scalability of the study's approach. Ethical considerations, such as phone retention, may also be under-discussed in terms of their potential impact on the findings or on the design of the study.*

Response: To address the generalizability of this research we added a section in lines 702-714 stating: "Our findings show that mobile applications using hardware on budget smartphones can generate useful data with limited interventions and suggests that mobile application development should be more widely considered in citizen science data collection efforts. Future research on the role of incentivization for data collection that utilizes technology should consider how to further disambiguate the inherent benefits in participation related to technological access, such as social status or monetary benefit from retaining equipment. We suggest that with increased mobile phone ownership, this complexity may be overcome by utilizing current phone owners, rather than providing participants with hardware."

The focus group discussions did not include specific questions that would allow us to analyse differences in culture and community norms beyond the motivations participants stated before the trial or their experiences afterwards. Differences in attitude and community norms were addressed in the discussion lines 575-678, and we have added in lines 709-712 a call for more crossectional work that may generate the data necessary to analyse the effects of infrastructure and community norms.

Comment 6: *Future Research Directions: the conclusion could benefit from a brief discussion of future research priorities, such as testing alternative incentive models, evaluating long-term engagement strategies, and/or assessing the usability of the acoustic mosquito sensors in the study context.*

Response: Future research directions we have added a section to the discussion in lines 702-713: "Future research on the role of incentivization for data collection that utilizes technology should consider how to further disambiguate the inherent benefits in participation related to technological access, such as social status or monetary benefit from retaining equipment. We suggest that with increased mobile phone ownership, this complexity may be overcome by utilizing current phone owners, rather than providing participants with hardware. Additionally, as mobile phone use becomes more popular, opportunities for large scale studies will support more complex statistical approaches to account for the complex structures that underpin intrinsic motivators and responses to extrinsic motivators, such as proximity to urban areas which are potentially obscured by the inferential statistical methods used in this study."

Comment 7*: Methodology improvement: the supplementary file provides detailed documentation of participant demographics and focus group questions, which supports the reproducibility of the study. Nevertheless, the modified random walk technique used for participant selection is not sufficiently explained in either the manuscript or the supplementary materials. While the inclusion of demographic comparisons and FGD protocols is quite valuable, the lack of detail about the recruitment method limits the transparency of the methodology. For instance, how were starting points for recruitment selected? Were any randomisation procedures applied to maintain the sampling unbiased? Additionally, minor gaps in the description of the design of text message reminders could also limit reproducibility. It is recommended that the authors provide additional detail or supplementary materials for these two aspects.*

Response: Details for how random walks were carried out is included in the methodology in lines 247-249, with details on reducing bias added in line 248: "To reduce the bias of entry point selection, each household was approached sequentially along the street and asked if they wanted to participate, until the number of households required to participate at the site (n=37) was met."

Regarding the methodological details of the SMS reminders, those reminders were not a component of this study but were part of the first study in Tanzania.

Comment 8: *Data availability seems to be in compliance with PLOS guidelines. Still, it is important to confirm that the repository cited by the authors includes raw data points (such as the individual participation records and anonymised FGD transcripts), as I was not able to access it. The statistical methods used appear to be appropriate, but I would recommend consulting someone with higher expertise in this matter. The methodology is sufficiently detailed overall. However, minor gaps in the description of participant recruitment (e.g., modified random walk technique) and the design of text message reminders could be further improved.*

Response: The additional raw data is available now at 10.6084/m9.figshare.27332124.

Comments from Reviewer 2

This study has addressed an important question about the influence of monetary incentives on participation (data collection) in citizen research. The methodology and results have been excellently presented and discussed. The researchers have done an excellent job of using quantitative and qualitative methods to address their study questions. The manuscript is indeed of very high quality.

Here are a couple of comments that I recommend that the authors consider:

Comment 1: *The topic of the study is not congruent with the study objective or question. The title "What incentives encourage local communities to collect and upload mosquito sound data by using smartphones? A case study in the Democratic Republic of the Congo" suggests that the study has evaluated different type of incentive while it has specifically assessed the influence of "monetary incentives" A title that would fit better with the study objective/question/scope could be "Do monetary incentives encourage local communities to collect and upload mosquito sound data by using smartphones? A case study in the Democratic Republic of the Congo"*

Response: The title of the manuscript has been adjusted to account for this suggestion.

Comment 2: *The researchers may have made efforts to adjust for and discuss confounding inherent on the research design but need to describe this more clearly in the report. Also, I recommend that authors highlight known limitations of the main inferential statistical approach used in comparative analysis.*

Response: A sentence has been added in the methods to address the use of post-hoc test to reduce risk of type 1 error in line 341.

To address the limitation of inferential statistics, a section has been added to the discussion in lines 709-712, suggesting future studies use cross-sectional data that may allow for more complex modelling approaches in analysis.

In addition to the above comments, all spelling and grammatical errors pointed out by the reviewers have been corrected. We look forward to hearing from you regarding our submission and to respond to any further questions and comments you may have.

Sincerely,

Kieran E. Storer

Kieran Stones

22/April/2025

Click here to download Data Review URL
http://figshare.com/articles/dataset/Raw_data_and_R_code_on_participa
nt_data_collection_in_study_in_the_DRC_characterizing_the_effects_of_
monetary_incentives_on_data_collection_/27332124
