# Supplementary material for: Do monetary incentives encourage local communities to collect and upload mosquito sound data using smartphones? A case study in the Democratic Republic of the Congo
Source: PLoS One. 2025 Aug 7;20(8):e0314122. doi: 10.1371/journal.pone.0314122 (PMC12331038; doi:10.1371/journal.pone.0314122)
Supplement: Attachment — Submitted filename: Response_to_Reviewers_auresp_2.docx [file pone.0314122.s006.docx]

Dear Dr. da Luz,

Thank you for the opportunity to submit a revised draft of our manuscript titled “*Do incentives encourage local communities to collect and upload mosquito sound data using smartphones? A case study in the Democratic Republic of the Congo*” to *PLOS One*. We appreciate the time you and the third reviewer of the paper have taken to provide valuable feedback and insightful comments. We have made changes to answer the questions of reviewer three and have added further information to provide clarity of the incentive used in the study and of the study system. Please find below the point-by-point responses and edits made to the paper in line with feedback.

## Journal Requirements:

***Comment 1:*** *Please review your reference list to ensure that it is complete and correct. If you have cited papers that have been retracted, please include the rationale for doing so in the manuscript text or remove these references and replace them with relevant current references. Any changes to the reference list should be mentioned in the rebuttal letter that accompanies your revised manuscript. If you need to cite a retracted article, indicate the article’s retracted status in the References list and also include a citation and full reference for the retraction notice.*

**Response**: The citation list has been reviewed and there were no instances of retracted papers. The URL links to some references were incomplete and these have now been amended. The reference list was amended with references to more completely explain the health administrative regions of the DRC and the geographic and population data of the country, with the previous references re-numbered to reflect this change.

43. United States Department of Agriculture, Foreign Agricultural Service. Congo: Democratic Republic of Congo - Country Overview. Nairobi: Global Agricultural Information Network; 2025 Feb 26. Report No.: KE2025-0004.

46. GRID3. GRID3 COD Health Areas v5.0 [Internet]. New York: Geo-Referenced Infrastructure and Demographic Data for Development (GRID3); 2023 [cited 2025 Jun 9]. Available from: <https://data.grid3.org/datasets/GRID3::grid3-cod-health-areas-v5-0/explore>

47. World Population Review. Kinshasa Population 2024 [Internet]. 2024 [cited 2025 Jun 6]. Available from: <https://worldpopulationreview.com/cities/dr-congo/kinshasa>

48. Institut National de la Statistique (INS), République Démocratique du Congo. Annuaire statistique 2020 [Internet]. Kinshasa: INS; 2021 [cited 2025 Jun 9]. Available from: UNDP‑CD mirror (PDF).

## Comments from Reviewer 3

***Comment 1:*** *My main concern is that the paper found "no effect of incentives" on outcomes. However, it is not clear from paper how incentivization was implemented and measured. yes, this is "key" in the paper. Therefore, the "no effect" reported in the paper might result from a flawed design and implementation of the "incentivization process". The authors should elaborate a bit more on that.*

**Response:** We were somewhat puzzled by this comment because the details of how incentivization was implemented are in manuscript lines 239-242 as follows: ‘*Control group participants were provided with the HumBug tool (a smartphone running the MozzWear app and the HumBug bed net), and one dollar to pay for an internet connection. The incentive group was provided with the HumBug tool and one dollar for an internet connection, and an additional ten dollars each month paid via airtime to their mobile phones.’* The incentive in this trial was the ten dollars paid each month*.* It remains unclear to us therefore what additional information reviewer 3 requires. We also note that the other two reviewers did not find this information on incentives lacking in the paper.

Similarly, the outcomes measured in the trial are detailed in lines 122-132: ‘*i) participant data collection activity during the trial period; ii) participant effort (the number of uploads per participant made during the sampling period, indicative of following trial protocols); and iii) the persistence of participation over time (whether trial participants continued to upload data throughout the sampling period). To address these questions, we compared participant activity (weeks active) and sampling effort (number of uploads) over the sampling period to assess differences in participation associated with receiving a monetary incentive for data collection. This study builds upon our previous work in Tanzania to fill gaps in understanding how monetary incentives alone influence the consistency and quality of audio data collection.’* We therefore remain unclear what more the reviewer would wish to see. Again, the other two reviewers did not find this information lacking. However, we have now added specification in line 130 that we are testing the effect of a monetary incentive on data collection to hopefully provide greater clarity.

***Comment 2:*** *Another thing, the paper is silent on how sample sizes in the treatment and control groups were calculated. This might support the "no effect" or explain the "no effect" observed in the paper.*

**Response:** Again, we are puzzled by this comment. Sample size calculations are clearly described in methodology line 247, stating the 37 households were recruited to participate at each site with lines 341-344 detailing sample size changes due to some participants dropping out of the study as follows: *‘Some participants decided to withdraw from the trial during its course and others failed to upload data, resulting in overall participation of 33 from the control group and 31 from the incentive group in the Kinshasa trial sites (n=64), and 33 from the control group and 35 from the incentive group in Bandundu trial sites (n=68), totaling 132 participants’*. These were the sample sizes used in statistical analysis. However, to further clarify, we have now also added repetition of sample sizes to figure legends, throughout the results, and in the supplement.

As requested by reviewer 3 we have now made it clear that Kinshasa is referred to as a province in this paper rather than as a city and we have further added clarifying details on the health administrative set up of the country and used this terminology throughout the manuscript.

We look forward to hearing from you regarding our submission and to respond to any further questions and comments you may have.

Sincerely,

Kieran E. Storer

13/June/2025
